# RNA structural dynamics regulate early embryogenesis through controlling transcriptome fate and function

**DOI:** 10.1186/s13059-020-02022-2

**Published:** 2020-05-18

**Authors:** Boyang Shi, Jinsong Zhang, Jian Heng, Jing Gong, Ting Zhang, Pan Li, Bao-Fa Sun, Ying Yang, Ning Zhang, Yong-Liang Zhao, Hai-Lin Wang, Feng Liu, Qiangfeng Cliff Zhang, Yun-Gui Yang

**Affiliations:** 1 CAS Key Laboratory of Genomic and Precision Medicine, Collaborative Innovation Center of Genetics and Development, College of Future Technology, Beijing Institute of Genomics, Chinese Academy of Sciences, Beijing, 100101 China; 2 University of Chinese Academy of Sciences, Beijing, 100049 China; 3 MOE Key Laboratory of Bioinformatics, Center for Synthetic and Systems Biology, Beijing Advanced Innovation Center for Structural Biology, Tsinghua-Peking Joint Center for Life Sciences, School of Life Sciences, Tsinghua University, Beijing, 100084 China; 4 State Key Laboratory of Membrane Biology, Institute of Zoology, Chinese Academy of Sciences, Beijing, 100101 China; 5 Institute of Stem Cell and Regeneration, Chinese Academy of Sciences, Beijing, 100101 China; 6 State Key Laboratory of Environmental Chemistry and Ecotoxicology, Research Center for Eco-Environmental Sciences, Chinese Academy of Sciences, Beijing, 100085 China

**Keywords:** RNA structure, Zebrafish early embryogenesis, Structure-based regulome, Elavl1a

## Abstract

**Background:**

Vertebrate early embryogenesis is initially directed by a set of maternal RNAs and proteins, yet the mechanisms controlling this program remain largely unknown. Recent transcriptome-wide studies on RNA structure have revealed its pervasive and crucial roles in RNA processing and functions, but whether and how RNA structure regulates the fate of the maternal transcriptome have yet to be determined.

**Results:**

Here we establish the global map of four nucleotide-based mRNA structures by icSHAPE during zebrafish early embryogenesis. Strikingly, we observe that RNA structurally variable regions are enriched in the 3′ UTR and contain *cis*-regulatory elements important for maternal-to-zygotic transition (MZT). We find that the RNA-binding protein Elavl1a stabilizes maternal mRNAs by binding to the *cis*-elements. Conversely, RNA structure formation suppresses Elavl1a’s binding leading to the decay of its maternal targets.

**Conclusions:**

Our study finds that RNA structurally variable regions are enriched in mRNA 3′ UTRs and contain *cis*-regulatory elements during zebrafish early embryogenesis. We reveal that Elavl1a regulates maternal RNA stability in an RNA structure-dependent fashion. Overall, our findings reveal a broad and fundamental role of RNA structure-based regulation in vertebrate early embryogenesis.

**Electronic supplementary material:**

Supplementary information accompanies this paper at 10.1186/s13059-020-02022-2.

## Background

Fertilization activates an array of developmental programs that transform a single-cell zygote into a multicellular organism. Embryogenesis in vertebrates is initially directed by maternal mRNAs deposited in the egg [1–4]. Before zygotic genome activation (ZGA), maternal transcripts undergo posttranscriptional and translational regulation, including selective elongation of the poly(A) tail [5], orchestrated translation [6, 7], and well-controlled clearance [1, 3]. Studies have started to unveil the regulatory program of this large-scale remodeling and function of maternal transcriptome. Protein synthesis was reported to be increased soon after fertilization in a selective manner [6, 7]. The poly(A) tails of maternal RNAs remain short immediately after fertilization, and then gradually elongate during the cleavage stage, which might coincide with translation activation [8]. During the maternal-to-zygotic transition (MZT) in zebrafish, miR-430 expressed from the zygotic genome triggers the degradation of hundreds of maternal RNAs [9]. Condon usage and 3′ UTR length have been shown to closely correlate with maternal RNA stability [10, 11], and the RNA modifications of m^6^A and m^5^C regulate maternal RNA stability [12, 13]. Beyond the discoveries of these individual pathways, the global mechanism for the regulatory program of maternal transcriptome however remains largely unknown.

RNA’s folding into intricate secondary structures is crucial for its function in vivo, and its misfolding is known to be associated with genetic disorders [14, 15]. Recent technological advances that couple chemical probing with high-throughput sequencing have enabled the measurement of RNA structures on the whole transcriptome scale, i.e., structuromes, in one experiment [16–20]. Studies in living cells are starting to reveal a once largely hidden layer of structure-based genetic regulation in nearly every step of RNA processing, including splicing, polyadenylation, localization, translation, and degradation [21–29]. Hence, RNA structure-based regulation is reasonably speculated to be essential in early embryogenesis. A recent study used dimethyl sulfate sequencing (DMS-seq) to profile the RNA structurome of two nucleotides (adenosine and cytosine) during 2–6 h post fertilization (h.p.f.) of zebrafish [30]. Here, we profiled the landscapes of four nucleotide-based RNA structure during zebrafish early embryogenesis at stages from 0 h.p.f. (fertilized egg) to 6 h.p.f. (shield) using our previously reported technique of icSHAPE (in vivo click selective 2′-hydroxyl acylation and profiling experiment) [19]. We demonstrated a previously unappreciated yet fundamental role of RNA structure-based regulation of the maternal transcriptome in the MZT.

## Results

### The RNA structure landscape during zebrafish early embryogenesis revealed “hot” structurally variable sites enriched with *cis*-regulatory elements

**Fig. 1:**
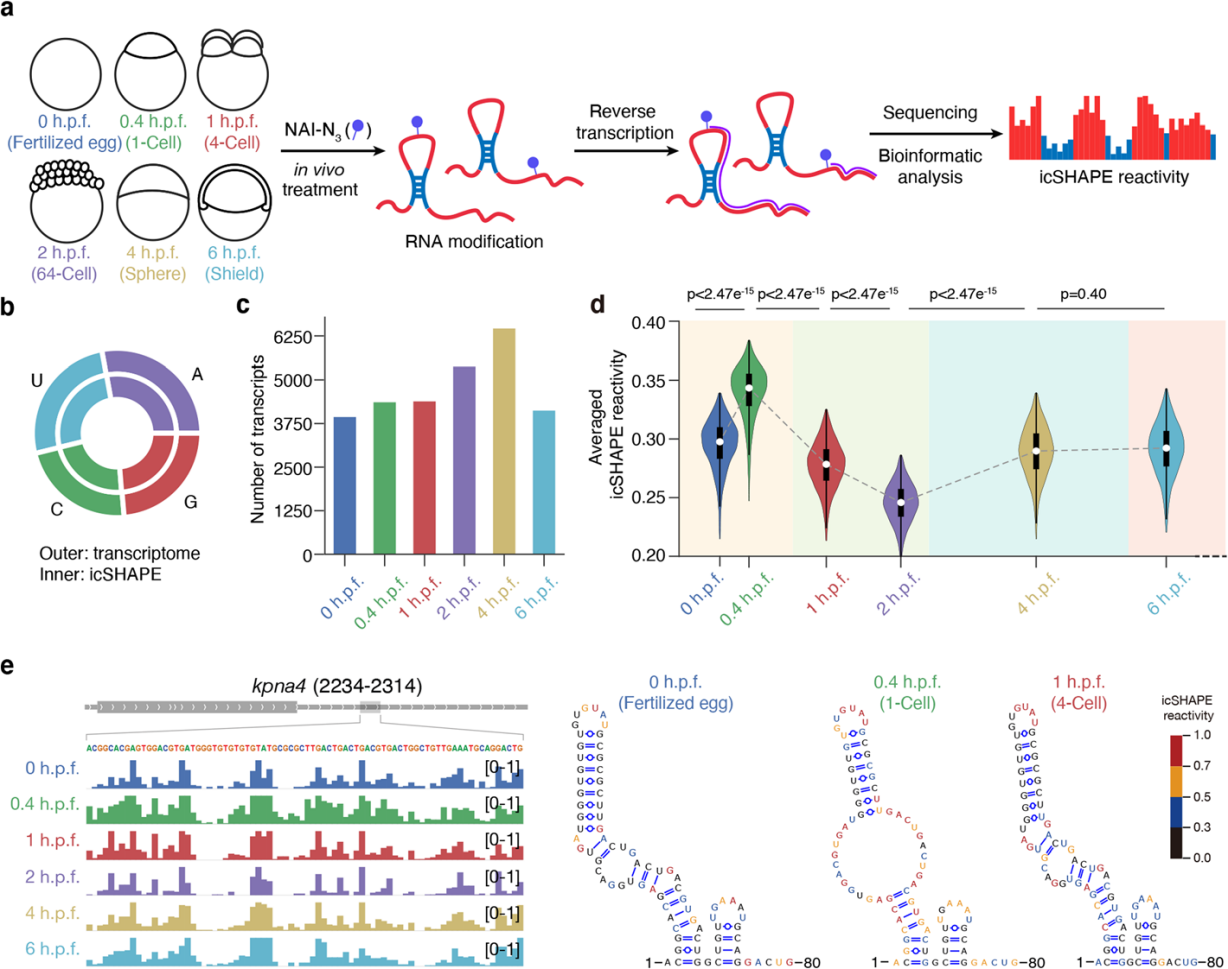
Comprehensive RNA structural maps during zebrafish early embryogenesis. **a** Schematic view of in vivo RNA structural maps during zebrafish early development using icSHAPE. **b** Nucleotide composition in transcriptome and all profiled sites (transcriptome, A: 27.91%, U: 25.96%, C: 22.47%, G: 23.66%; icSHAPE, A: 27.86%, U: 25.99%, C: 22.37%, G: 23.78%). **c** The number of transcripts with more than half of the nucleotides with valid structural signals at each stage. **d** Global structural changes by violin plot of average icSHAPE reactivity of each transcript during zebrafish early development; *P* values were calculated by paired two-sided Student’s *t* test. **e** Integrative Genomics Viewer (IGV) view of icSHAPE reactivity and RNA structure model of *kpna4* gene at 3′ UTR region

To characterize the RNA structuromes in zebrafish early embryogenesis, we performed in situ icSHAPE [19], an unbiased high-throughput technique potently measuring the structural flexibility of every nucleotide, in zebrafish early embryos at stages of 0 h.p.f. (fertilized egg), 0.4 h.p.f. (1-cell), 1 h.p.f. (4-cell), 2 h.p.f. (64-cell), 4 h.p.f. (sphere), and 6 h.p.f. (shield) (Fig. 1a; Additional file 1: Fig. S1a, b). We determined the RNA structures as previously described [19] and obtained transcriptome-wide RNA structural maps with a high correlation between biological replicates at both transcript (Additional file 1: Fig. S1c) and nucleotide levels (Additional file 1: Fig. S1d). Analysis of conserved secondary structures of *dgcr8* [31] showed that paired bases exhibit lower icSHAPE reactivity than single-stranded regions (*P* value = 2.5 × 10^− 9^). The receiver operating characteristic (ROC) curve was then plotted to evaluate the concordance between icSHAPE reactivities and the conserved secondary structures of *dgcr8*. A high agreement between the icSHAPE reactivity with the conserved secondary structure was observed, and the area under curve (AUC) was positively associated with the agreement (Additional file 1: Fig. S1e-g). Overall, about 4000 transcripts were obtained in each of developmental stages, with concordant coverage for all four nucleotides in the transcriptome and icSHAPE-treated RNAs (Fig. 1b, c; Additional file 1: Fig. S1h; Additional file 2). In sum, we established the all-4-base RNA structural landscape in early developmental stages at an unprecedented depth and coverage.

We found that the RNA structuromes are highly dynamic from 0 h.p.f. (fertilized egg) to 6 h.p.f. (shield), with a structural opening immediately after fertilization and a gradual close until 2 h.p.f. (64-cell) followed by a reopening (Fig. 1d). To check whether these structural changes are due to differential RBP binding across different developmental stages, we performed in vivo mRBPs (mRNA-binding proteins) pulldown by using 0/0.4/4 h.p.f. embryos (Additional file 3). We did not observe any changes of global mRBP binding among these stages (Additional file 1: Fig. S1i). We further systematically analyzed the icSHAPE reactivity score distribution in a very large dataset of RBP binding sites of 23 iCLIP studies in zebrafish [32], and compared it with that of the random nucleotides on the whole transcripts. If RBP binding generally protects RNA from the probing reaction of the icSHAPE reagent NAI-N_3_, the icSHAPE reactivity scores would be lower at these RBP binding sites when compared to random positions. However, only very small reactivity score differences between these two datasets were observed, suggesting that RBP binding does not necessarily block NAI-N_3_ modification (Additional file 1: Fig. S1j). Figure 1e and Additional file 1: Fig. S1k show dynamic changes observed in the RNA structures of *kpna4* mRNA by Integrative Genomics Viewer (IGV).

The previous study by Beaudoin et al. showed that translation drives ORF structural opening [30]. The structural data in our study by icSHAPE also found that the translation efficiency correlates with mRNA accessibility in zebrafish embryos (Additional file 1: Fig. S2a), indicating an interplay between RNA structure and translation during zebrafish embryogenesis.

**Fig. 2:**
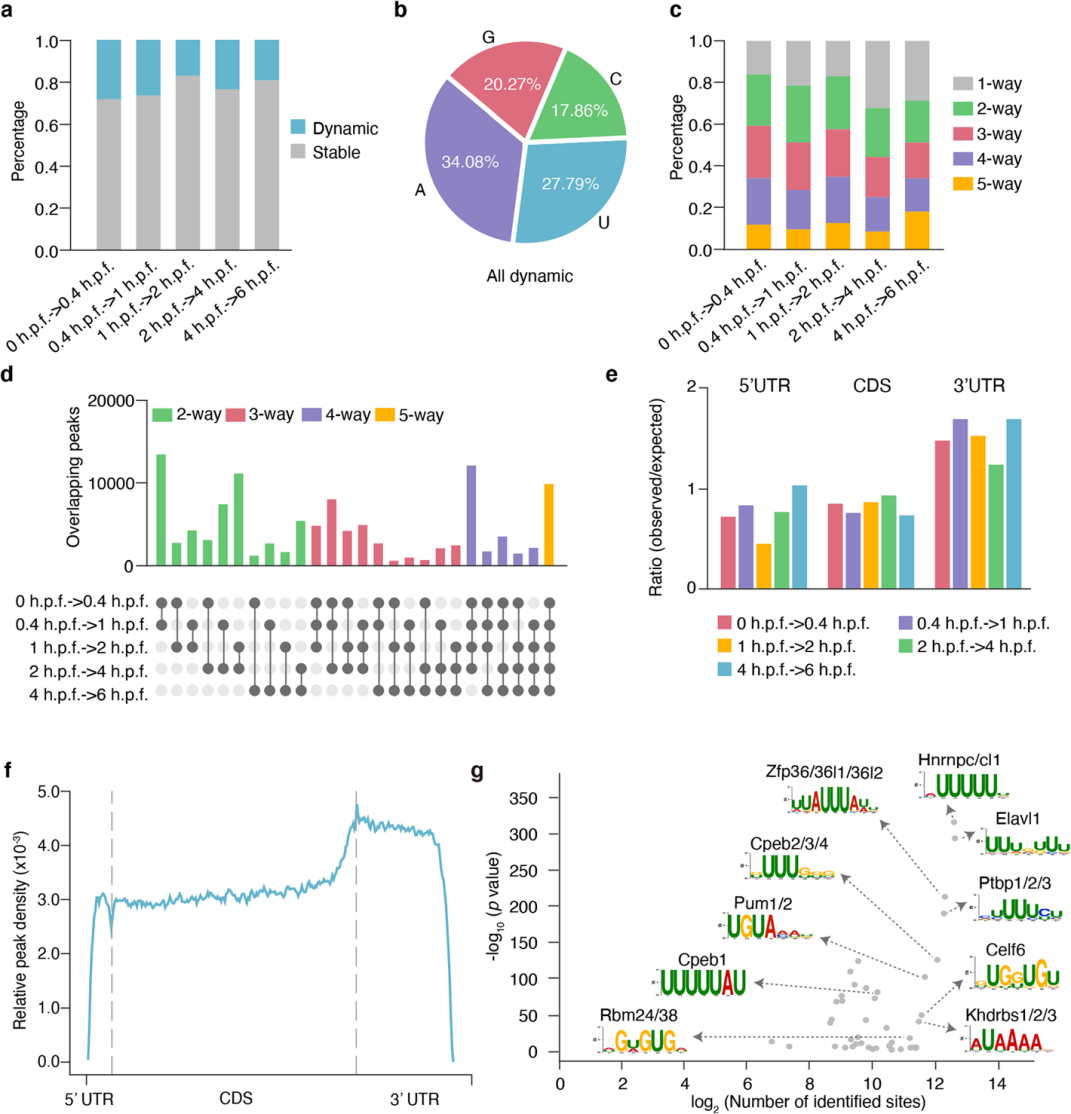
Hot structurally variable sites enriched with *cis*-regulatory elements. **a** Distribution of structurally variable nucleotides between adjacent developmental stages. **b** Nucleotide composition of all structurally variable nucleotides. **c** The distribution of common (2,3,4,5-way) or specific (1-way) structurally variable regions in different comparisons. The structurally variable region that is specific to one comparison is termed as “1-way,” while 2-way means that the structurally variable regions is shared by two comparisons, 3-way means that it is shared by three comparisons, and so on. **d** The statistics of all structurally variable regions grouped by their commonality. **e** Ratio of observed counts and expected counts of all structurally variable windows in three segments: 5′ UTR, CDS, 3′ UTR. The ratio is calculated by observed counts divided by expected counts. Statistical significance of enrichment of structurally variable windows in 3′ UTR was carried out with Fisher’s exact; *P* value < 2.23 × 10^− 308^ for all comparisons. **f** Metagene profile depicts the sub-transcript distribution pattern of common structurally variable regions shared by at least two comparisons. **g** Scatter plot shows the significance and occurrences of RNA-binding motif enriched in common structurally variable regions at 3′ UTR shared by at least two comparisons. *P* values were calculated by Fisher’s exact test. Refer to method section “De novo motif discovery and enrichment analysis of structurally variable regions”

We designed a computational pipeline to analyze the composition and functional implication of these structurally variable regions. First, we chose the same cutoff of 0.2 as used in our previous study [33] to distinguish a base with or without differential icSHAPE reactivity score, and further performed icSHAPE on folded spike-in RNAs. We found that over 98.5% bases present differential icSHAPE reactivity score lower than 0.2 between replicates (Additional file 1: Fig. S2b). These results suggest that the cutoff of Δreactivity score > 0.2 is enough for filtering out most of the technical noise and, thus, can be used to assess the significant structural change for a single nucleotide. For 10-nt sliding windows, the similar cutoff strategy was used. Almost 95% 10-nt sliding windows had the differences lower than 0.05 in icSHAPE reactivity score (Additional file 1: Fig. S2c). In addition to the average Δreactivity score > 0.05, a *P* value less than 0.05 (two-sided paired Student’s *t* test) was also included for selecting the significant structural change for a 10-nt sliding window. Briefly, we identified structurally variable nucleotides that show a significant structural change at the cutoff of Δ_reactivity score_ > 0.2. Overall, about 18–28% of the nucleotides were defined as structurally variable across different developmental stages (Fig. 2a). We found that these nucleotides are enriched with adenosine and uracil (compare Fig. 2a with Fig. 1b, *P* value < 2.23 × 10^− 308^ Fisher’s exact test), which is consistent with previous observations that adenosine and uracil are structurally more flexible nucleotides when comparing RNA structures across different cellular compartments [33] as well as among samples under in vivo and in vitro conditions [19], suggesting their involvement in structure regulation. We also identified structurally variable regions by scanning the transcriptome with a 10-nt sliding window, and defined those regions with the average Δ_reactivity score_ > 0.05 and *P* value < 0.05 (two-sided paired Student’s *t* test) between two developmental stages as *structurally variable sites* (see “Methods”). We found that many variable regions are shared among different comparisons (Fig. 2c, d), suggesting that these sites are hot regions with structural changes. Interestingly, we observed that RNA structurally variable regions are enriched in the 3′ UTRs (Fig. 2e; Additional file 1: Fig. S2d, e; *P* value < 2.23 × 10^− 308^ for all comparison, Fisher’s exact test), especially for the shared structurally variable regions (Fig. 2f; Additional file 1: Fig. S2f).

We then searched for the signatures of enriched sequence within the 3′ UTR hot regions. Interestingly, the sequences contain many known binding sites of RNA-binding proteins (RBPs) that are functionally posttranscriptional regulators (Fig. 2g; Additional file 1: Fig. S2g; Additional file 4; Additional file 5), including those for RNA splicing, polyadenylation, translation, and stability. For example, 8.72% of the regions contain the cytoplasmic polyadenylation element binding protein (Cpeb1, motif: UUUUUAU), which is known to regulate embryonic cytoplasmic mRNA polyadenylation [34, 35]. About 4.64% of the regions contain the binding site motif of Pumilio (motif: UGUAAAU), which regulates translation and stability of maternal mRNAs through sequence-specific association with the 3′ UTRs during MZT [36, 37]. In addition, 16.45% of the regions contain the binding site motif of Elavl1 (motif: UUUGUUU) which had been reported to regulate mRNA stability [38, 39]. We analyzed all the transcripts with structurally variable regions in the 3′ UTRs that contain the DLE structure motif (a consensus “AGCAC” sequence in a hairpin/stem-loop in 3′ UTR, [40, 41]) (Additional file 6). We found that the genes are enriched with the functions of protein localization, transport, etc. (Additional file 1: Fig. S2h; Additional file 6). This finding is consistent with a previous study [41]. Furthermore, it could help to discover the potential protein regulators and understand how RNA structural changes participate in the regulation. In total, our analysis uncovered 50 binding site motifs for 98 proteins that span about 66.50% of these shared 3′ UTR hot regions, suggesting a large-scale structure-based regulome for the posttranscriptional regulation during zebrafish early embryogenesis. Intriguingly, most of the enriched binding motifs in structurally variable regions were U-rich. In contrast, DMS-seq can only probe two bases of A and C, which impairs its power to discover structurally regulated *cis*-elements.

### Elavl1a is enriched in variable structural regions in 3′ UTRs and prefers to bind single-stranded RNA in vivo and in vitro

**Fig. 3:**
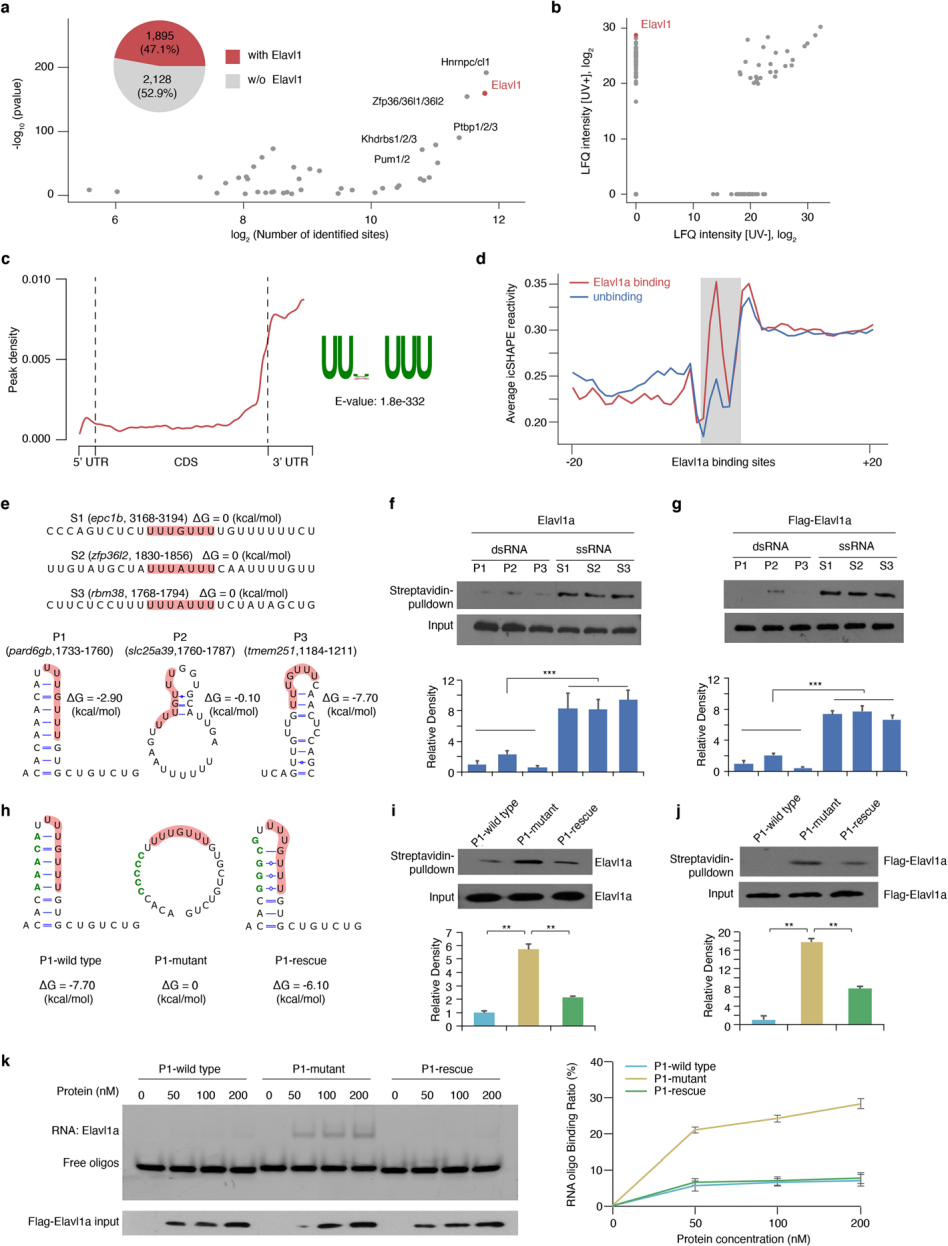
Elavl1a prefer to bind single-stranded RNA in vivo and in vitro which enriched in structurally variable regions in 3′ UTRs. **a** Scatter plot shows the significance and occurrence of RNA-binding motif enriched in structurally variable windows at 3′ UTR between 4 h.p.f. and 6 h.p.f.; *P* values were calculated by Fisher’s exact test. Inner pie chart shows 47.1% of transcripts with structurally variable regions at their 3′ UTR containing Elavl1 binding motif. **b** Scatter plot shows Elavl1a’s enrichment in UV (+) sample at 4 h.p.f.. LFQ, label free quantitation. **c** Distribution of Elavl1a peaks across the length of mRNA and binding motif identified by Dreme (MEME suite) with Elavl1a-binding peaks in 3′ UTR (*E*-value = 1.8 × 10^− 332^). **d** icSHAPE metaprofile around Elavl1a binding sites and unbound sites with the same motif shows that Elavl1a tend to bind ssRNA in vivo*.***e** The structure models of six endogenous RNA probes containing Elavl1a binding sites. Elavl1a binding sites were colored in red background. **f** Demonstration of endogenous Elavl1a pulled down by endogenous RNA probes containing Elavl1a binding sites. Upper, western blotting; lower, quantification level. Error bars, mean ± s.d., *n* = 3. *P* values were calculated using Student’s *t* test. **g** Demonstration of purified Flag-Elavl1a pulled down by endogenous RNA probes containing Elavl1a binding sites. Upper, western blotting; lower, quantification level. Error bars, mean ± s.d., *n* = 3. *P* values were calculated using Student’s *t* test. **h** The structure models of designed P1 wild-type, P1 mutant, and P1 rescue RNA probes containing Elavl1a binding sites and flanking regions. **i** Demonstration of endogenous Elavl1a pulled down by designed endogenous RNA probes containing Elavl1a binding sites. Upper, western blotting; lower, quantification level. Error bars, mean ± s.d., *n* = 3. *P* values were calculated using Student’s *t* test. **j** Demonstration of purified Flag-Elavl1a pulled down by designed endogenous RNA probes containing Elavl1a binding sites. Upper, western blotting; lower, quantification level. Error bars, mean ± s.d., n = 3. *P* values were calculated using Student’s *t* test. **k** EMSA (left) and line graph quantification (right) showing the binding ability of purified Flag-Elavl1a with designed P1 wild-type, P1 mutant, and P1 rescue RNA probes containing Elavl1a binding sites. In total, 100 nM of RNA probes was incubated with different concentrations of Flag-Elavl1a protein. The RNA binding ratio was calculated by (RNA protein) / ((free RNA) + (RNA protein)). Error bars, mean ± s.d., *n* = 3

Based on the above results, we speculated that RNA structure might regulate maternal RNA degradation. We therefore repeated the computational screening for *cis*-regulatory sequence-and-structure elements that could possibly regulate maternal RNA degradation. We performed a motif search in the structurally variable regions during 4 to 6 h.p.f. since the majority of maternal transcripts are degraded rapidly during this period [5]. The screening results revealed that about half (1895/4023) of the structurally variable transcripts contain binding motif of Elavl1, a previously reported mRNA stabilizing factor [38, 39], in their structurally variable regions (Fig. 3a; Additional file 5). In addition, we found that zebrafish Elavl1 displayed a high mRNA binding affinity in 4 h.p.f. stage embryos in mass spectrometry experiments (Fig. 3b; Additional file 3).

The previous work [42] had identified two *elavl1* gene orthologs in zebrafish, *elavl1a* and *elavl1b*, with the former one being more similar to human *ELAVL1* in genomic structure. To further explore the function of Elavl1 in early embryogenesis, we examined the expression of homologous genes in zebrafish embryos. The result showed that the expression level of *elavl1a* was much higher than that of *elavl1b*, suggesting that *elavl1a* is likely the dominant homologous gene during zebrafish early development (Additional file 1: Fig. S3a). Whole-mount in situ hybridization (WISH) analysis demonstrated that *elavl1a* mRNA is ubiquitously expressed (Additional file 1: Fig. S3b), and western blotting analysis showed the Elavl1a protein expression beginning at around 2 h.p.f. (Additional file 1: Fig. S3c). These findings led us to define the regulatory role of *elavl1a* in zebrafish early embryogenesis.

An individual-nucleotide resolution crosslinking and immunoprecipitation (iCLIP) assay was used to analyze the in vivo targets and structural preference of Elavl1a in zebrafish embryos (Additional file 1: Fig. S3d; Additional file 7), and the results showed that Elavl1a mainly binds to 3′ UTR by recognizing a U-rich motif in zebrafish (Fig. 3c). Through comparing with human ELAVL1 binding targets [39, 43], over 60% of Elavl1a binding genes identified in zebrafish are also targeted by ELAVL1 in human (Additional file 1: Fig. S3e). Two Elavl1a binding targets in zebrafish were exemplified by IGV (Additional file 1: Fig. S3f).

We then performed meta-analysis on the structural profile at Elavl1a binding sites detected by iCLIP, and the results showed that the sites with Elavl1a binding are more accessible than the nonbinding ones even though they have the same sequence motif, suggesting that Elavl1a tends to bind single-stranded RNAs in vivo (Fig. 3d).

To explore whether or not in vivo Elavl1a expression influences the RNA structure at Elavl1a binding sites, we performed icSHAPE assays by using wild-type (WT) and Elavl1a knockdown samples at stages of 4 h.p.f. and 6 h.p.f.. Elavl1a knockdown was carried out by *elavl1a*-targeted ATG morpholino (MO) injection for translation inhibition (Additional file 1: Fig. S3g), which was verified by western blotting (Additional file 1: Fig. S3h). The metagene analysis of the structural profile at Elavl1a binding sites showed that these binding sites are more accessible than the nonbinding sites with the same sequence motif, even in the condition of Elavl1a deficiency (Additional file 1: Fig. S3i, j).

To further examine whether Elavl1a prefers to bind single-strand RNA (ssRNA) or double-strand RNA (dsRNA), biotin-labeled RNA pulldown assays were carried out by six probes containing endogenous Elavl1a binding sites (Fig. 3e). The results of both in vivo and in vitro RNA pulldown assays demonstrated that the Elavl1a protein can be more efficiently pulled down by ssRNA oligonucleotides relative to dsRNA oligonucleotides (Fig. 3f, g). Consistently, electrophoretic mobility shift assays (EMSA) (Additional file 1: Fig. S3k) also illustrated that Elavl1a prefers to bind ssRNA instead of dsRNA.

To test whether RNA structure sufficiently influences Elavl1a’s binding ability, we repeated the above analyses using different structural conformations of biotin-labeled probes containing an endogenous Elavl1a binding site, including P1-wild-type with partially base paired Elavl1a binding site, P1-mutant with disrupted base pairing at the binding site, and P1-rescue with restored base pairing at the binding site (Fig. 3h). The findings from both in vivo and in vitro pulldown (Fig. 3i, j) and EMSA (Fig. 3k) assays illustrated that Elavl1a had a higher binding ability to ssRNA oligonucleotide than to dsRNA oligonucleotide. Thus, RNA structure could efficiently affect Elavl1a’s binding ability.

### RNA structurally variable elements in Elavl1a binding regions correlate with maternal RNA stability

**Fig. 4:**
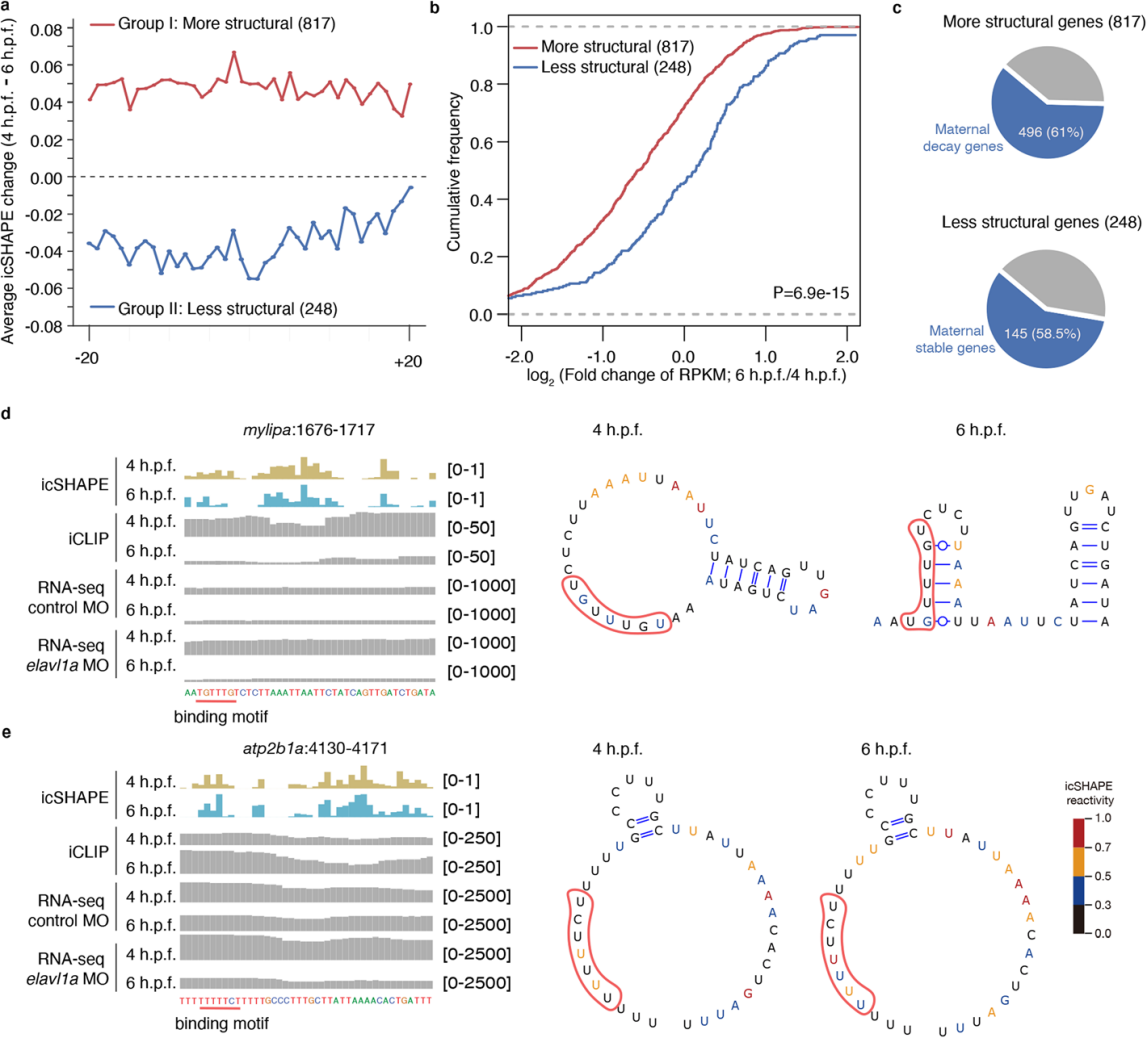
RNA structurally variable elements in Elavl1a binding regions associate with maternal RNA stability. **a** Average icSHAPE reactivity change (4 h.p.f. and 6 h.p.f.) at the Elavl1a binding site of the two groups of transcripts. Group I: more structural, forming more structure at Elavl1a binding sites. Group II: less structural, forming less structure at Elavl1a’s binding sites. “More structural” group was defined as the average of icSHAPE reactivity of those Elavl1-binding sites at 6 h.p.f. is less than that at 4 h.p.f. and the differences were larger than 0.05 (lower icSHAPE reactivity indicates that RNA structure become less accessible to SHAPE reagent, thus become more compact structure). Statistical significance was calculated by paired *t*-test and set to be *P* < 0.05. While the “less structural” group is defined as the average of icSHAPE reactivity of those Elavl1-binding sites at 6 h.p.f., it was larger than that at 4 h.p.f. and the difference is also larger than 0.05, *P* < 0.05. **b** Cumulative distribution of the log_2_ fold changes of the RNA level between two group transcripts with more or less structural Elavl1a binding sites during the period of 4 h.p.f. to 6 h.p.f.. *P* value was calculated using two-sided Wilcoxon test. **c** Pie chart depicting the proportion of maternal decay genes in transcripts with more structural Elavl1a binding sites, and the proportion of maternal stable genes in transcripts with less structural Elavl1a binding sites during the period of 4 h.p.f. to 6 h.p.f.. **d, e** IGV tracks and structure profile displaying icSHAPE (upper panels), iCLIP-seq (middle panel), and RNA-seq (bottom panel) read distributions in 3′ UTR of *mylipa* (**d**) and *atp2b1a* (**e**) mRNA. Binding motifs are indicated with red highlight

We further examined the potential effects of RNA structural changes on Elavl1a’s functionality in maternal RNA regulation. Two groups of RNAs were defined by forming more (group I, Fig. 4a, upper) or less RNA structure (group II, Fig. 4a, lower) in Elavl1a binding regions between 4 h.p.f. and 6 h.p.f.. Through comparing their transcript abundance changes, transcripts in group I were observed to be significantly downregulated relative to group II (Fig. 4b, *P* value = 6.9 × 10^− 15^, two-sided Wilcoxon test), suggesting that potency of Elavl1a in stabilizing RNAs is substantially influenced by the structural shift in its binding regions, and moreover, Elavl1a stabilizes its target transcripts when its binding sites within single-stranded regions are easily accessible. Through comparing the gene expression levels between 2 and 6 h.p.f., we categorized the genes into three groups: (i) genes with RPKM > 1 at 2 h.p.f., log_2_(FC) > log_2_(1.2), and FDR < 0.05 were defined as maternal decay genes; (ii) genes with RPKM > 1 at 2 h.p.f., log_2_(FC) < log_2_(1.2), and FDR < 0.05 were defined as maternal stable genes; and (iii) genes with RPKM < 1 at 2 h.p.f. and RPKM > 1 at 6 h.p.f. were defined as zygotic genes (Additional file 8).

About 61% (496/817) of group I transcripts belong to the maternal decay genes (Fig. 4c upper; Additional file 1: Fig. S4a upper), whereas about 59% (145/248) of group II transcripts belong to the maternal stable genes (Fig. 4c lower; Additional file 1: Fig. S4a lower). We also validated this finding in zebrafish early embryogenesis by RNA immunoprecipitation-qPCR (RIP-qPCR), and the findings showed that Elavl1a’s binding ability to the targets was markedly decreased at 6 h.p.f. owing to more structures formed at Elavl1a’s binding sites (Additional file 1: Fig. S4b). For instances, *mylipa* (Fig. 4d) forms more structure at Elavl1a’s binding sites during 4 h.p.f. to 6 h.p.f., which excludes the Elavl1a binding and consequently results in its rapid degradation. In contrast, *atp2b1a* (Fig. 4e) allows the easily accessibility for Elavl1a to its binding sites, leading to the enhanced Elavl1a binding and RNA stability.

To define maternal decay/stable genes more reliably, we intersect our maternal gene set with the maternal and paternal transcriptomes reported by Harvey et al. [44]. We found that 3182 genes from our gene set could distinguish maternal or zygotic expression by using maternal vs. paternal SNPs. Among these, 859 genes were only maternally expressed, 143 genes were only zygotically expressed, and 2180 genes had both maternal and zygotic expression (Additional file 8). In particular, 94.3% (810/859) of the maternal-only genes decayed between 2 h.p.f. and 6 h.p.f., and the stable ones accounted for only 4.9% (42/859) (Additional file 1: Fig. S4c). In contrast, for the genes with both maternal and zygotic expression, 65.3% (1424/2180) were decayed, while 32.6% (710/2180) were stable between 2 h.p.f. and 6 h.p.f. (Additional file 1: Fig. S4d). Thus, even accounting for the zygotic contributions, most of these genes still showed a decreased expression during zebrafish embryogenesis. After narrowing down the definition of maternal gene set by maternal vs. paternal SNPs, 274 out of 817 group I genes were categorized into the “more structural” group and 69% (187/274) showed maternal decay, with 24 and 163 in maternal-only and maternal-and-zygotic group, respectively (Additional file 1: Fig. S4e, left). However, in the “less structural” genes, 62% (38/61) were maternally stable with 1 and 37 in the maternal-only and maternal-and-zygotic groups, respectively (Additional file 1: Fig. S4e, right). These results are very similar to the findings obtained from the much bigger maternal gene set defined by gene expression change.

We further analyzed the structural changes at the Elavl1a binding sites forming more structures between 4 h.p.f. and 6 h.p.f.. A similar change tendency was observed between the wild-type and Elavl1a-deficiency embryos, suggesting that these changes are independent of the Elavl1a binding (Additional file 1: Fig. S4f). Taken together, these results suggest that it is more likely that the RNA structural changes are not due to Elavl1a binding, but originate from an “RNA structure-switch” and modulate the protein binding.

### Elavl1a-mediated mRNA stability is required for early development

**Fig. 5:**
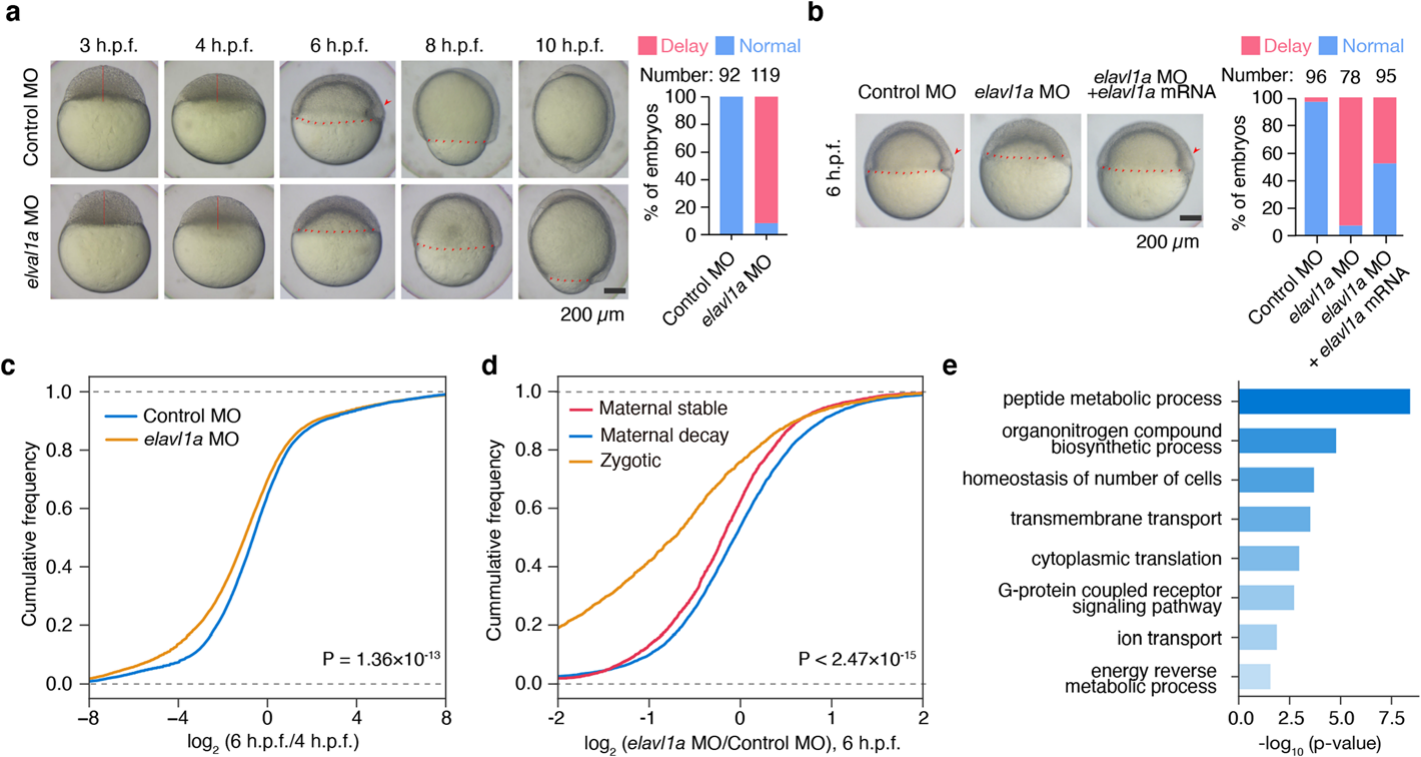
Developmental delay and accelerated maternal RNA clearance induced by Elavl1a deficiency. **a** Elavl1a deficiency leads to developmental delay during zebrafish early embryogenesis. **b** Zebrafish *elavl1a* mRNA with MO-mismatch binding site can partially rescue the phenotype in *elavl1a* morphants. **c** Cumulative distribution of the log_2_ fold changes of RNA level between control and *elavl1a* morphants during the period of 4 h.p.f. to 6 h.p.f.. *P* values were calculated by two-sided Kolmogorov-Smirnov test. **d** Cumulative distribution of the log_2_ fold changes of RNA level of *elavl1a* morphants versus control in maternal decay, maternal stable and zygotic groups at 6 h.p.f.. *P* values were calculated by two-sided Kolmogorov-Smirnov test. **e** Gene set enrichment analysis of downregulated genes upon Elavl1a deficiency

To determine if Elavl1a is functionally required for zebrafish embryogenesis, we knockdown Elavl1a by *elavl1a* MO injection and examined the early development. Both the control and *elavl1a* morphants (MO injected embryos) could reach the 1k-cell stage through completing cell cycle 10 at 3 h.p.f.. However, the transition from the 1k-cell stage to sphere was moderately postponed, and the subsequent gastrulation was markedly delayed in *elavl1a* morphants (Fig. 5a). In addition, *elavl1a* morphants displayed various malformations, such as decreased head size, curved anterior-posterior axis, and shorter body length at 24 h.p.f. (Additional file 1: Fig. S5a). Importantly, this developmental delay can be partially rescued by injection of *elavl1a* mRNA with MO-mismatched binding site (Fig. 5b).

To further demonstrate the Elavl1a-deficiency phenotype, we generated *elavl1a*-null frameshift mutant by CRISPR/Cas9 technology and obtained zygotic mutant embryos (*elavl1a*^−/−^) by cross-mating heterozygous mutants (*elavl1a*^+/−^) (Additional file 1: Fig. S5b). However, we could not obtain *elavl1a* maternal mutant embryos by mating zygotic mutants because all the zygotic mutant individuals died before adulthood. Alternatively, we crossed heterozygous females with wild-type males to obtain progenies with reduced maternal *elavl1a* transcripts and then injected with low-dose of *elavl1a* MO to suppress the translation of residual maternal transcripts. Consistently, relative to wild-type embryos, the embryos obtained from crossing of heterozygous females and wild-type males exhibited obvious developmental delay after *elavl1a* MO injection (Additional file 1: Fig. S5c). Therefore, our evidence demonstrate that Elavl1a-deficiency causes developmental defect. Of note, the development is normal before the mid-blastula transition (MBT, about 3 h.p.f.) in Elavl1a*-*deficient embryos, suggesting that Elavl1a mainly regulates the developmental process post the rapid synchronous cell division period.

To further define the role of Elavl1a in a transcriptome-wide scale, we performed RNA sequencing at 4 and 6 h.p.f. in control and *elavl1a* morphants (Additional file 1: Fig. S5d). Elavl1a deficiency resulted in the accelerated degradation of mRNAs between 4 and 6 h.p.f. (Fig. 5c, *P* value = 1.36 × 10^− 13^, two-sided Kolmogorov-Smirnov test), suggesting a protective role of Elavl1a during MZT. Consistently, Elavl1a deficiency accelerated the degradation of maternal stable RNAs to a greater extent than the maternal decay ones (Fig. 5d, *P* value = 2.47 × 10^− 15^, two-sided Kolmogorov-Smirnov test). We also found that zygotic gene expression is also downregulated upon Elavl1a deficiency, which is probably owing to the delayed ZGA. Further GO analysis showed that downregulated transcripts are enriched in the processes of protein metabolism, cell homeostasis, transmembrane transport, and energy metabolism (Fig. 5e; Additional file9). These results suggest that Elavl1a plays an essential role in early development through stabilizing mRNAs required for MZT.

### Elavl1a regulates maternal RNA stability in a structure-dependent fashion

**Fig. 6:**
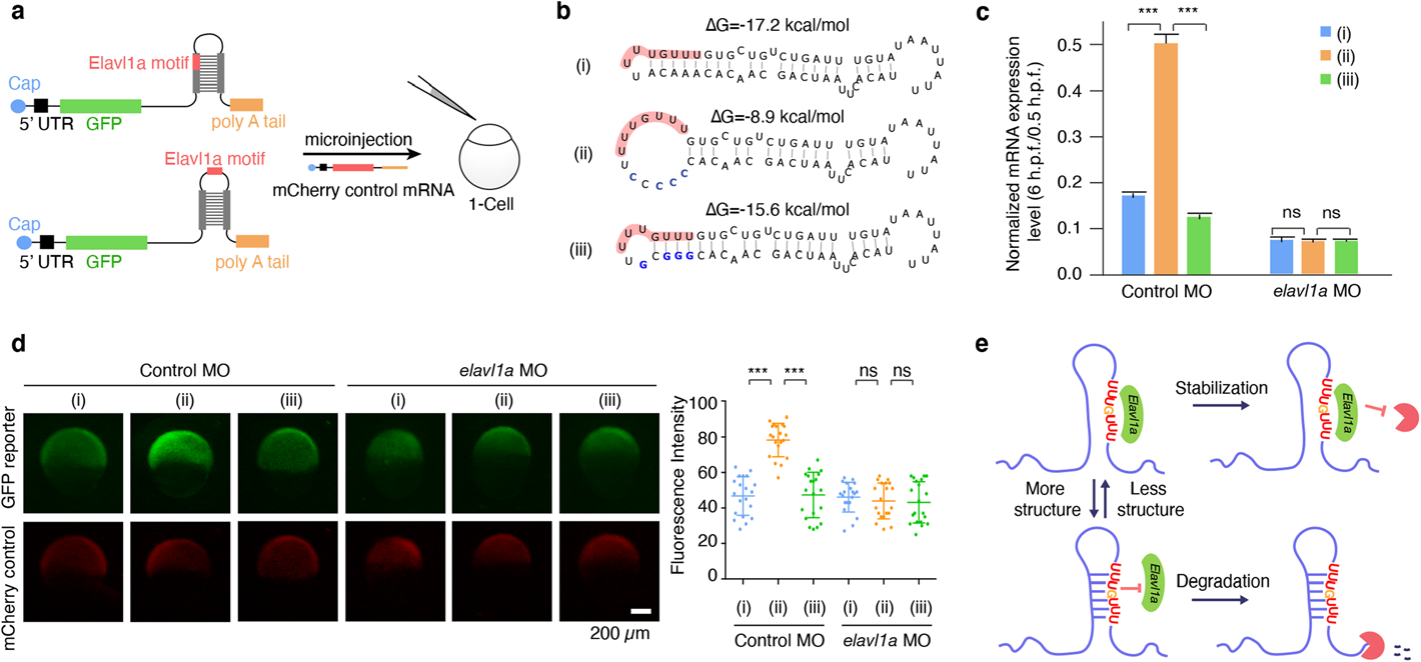
Elavl1a regulates maternal RNA stability in a structure-dependent fashion. **a** Design of the GFP reporter mRNA with single-stranded or double-stranded Elavl1a binding site in its 3′ UTR. **b** The structure models of designed (i) wild-type, (ii) mutant, and (iii) rescue Elavl1a binding sites and flanking regions. **c** The relative mRNA level (6 h.p.f. versus 0.5 h.p.f.) of reporter genes with different structural contexts of Elavl1a binding motif in control and *elavl1a* morphants, *n* = 3 for each group, error bars, mean ± s.d.; *P* values were determined by two-sided Student’s *t* test. **d** The protein level of reporter gene with different structural contexts of Elavl1a binding motif at 6 h.p.f. in control and *elavl1a* morphants determined by GFP fluorescence signal observation. Some pictures of representative embryos were shown. Quantitative fluorescence intensity was shown on the right, *n* = 20 for each group. Error bars, mean ± s.d. *P* values were determined by two-sided Student’s *t* test. **e** Schematic model shows that Elavl1a regulates RNA stability in a structure-dependent fashion

To test whether or not RNA structure is sufficient to influence Elavl1a-modulated mRNA stability, we constructed green fluorescent protein (GFP) reporter mRNAs, which are derived from partial endogenous 3′ UTR (*pard6gb*) containing Elavl1a binding site with different structural contexts, (i) the wild-type in which part of the Elavl1a binding site is base paired, (ii) a mutation that disrupts base pairing of the binding site, and (iii) a rescue that restores the base pairing (Fig. 6a, b). The mCherry mRNA was used as control. These GFP reporter mRNAs and mCherry control mRNA were injected into control or *elavl1a* morphants at 1-cell stage and their decay kinetics were examined by RT-qPCR. The results showed that the double-stranded flanking sequence GFP reporter mRNA was degraded faster than that of the single-stranded one during MZT in control group (Fig. 6c). In contrast, no difference was observed in the *elavl1a* morphants (Fig. 6c). Additionally, GFP protein expression was higher in control harboring single-stranded flanking sequence reporter mRNA compared to double-stranded ones (Fig. 6d). Similarly, no difference was identified in the *elavl1a* morphants (Fig. 6d). We further performed gene reporter assay using the full-length endogenous 3′ UTR sequence and obtained the similar results as partial endogenous 3′ UTR sequence (Additional file 1: Fig. S6a, b).

These observations clearly indicate that the RNA structure regulates the mRNA stability and consequent protein synthesis in zebrafish early embryogenesis through affecting Elavl1a’s accessibility to its binding motif. In conclusion, we proposed a model depicting the overall role of structurally variable changes of Elavl1a’s binding regions in regulating RNA stability through affecting Elavl1a’s binding ability to its mRNA targets (Fig. 6e).

## Discussion

Vertebrate early embryogenesis is characterized by the conversion of the maternal oocyte state to the embryonic totipotency via the involvement of a series of programmed genetic and epigenetic events for genome remodeling, such as DNA demethylation, chromatin remodeling, and spatial genome reorganization [45]. RNA structure is tightly involved in a myriad of posttranscriptional regulation on a global scale [22]. Analyses of RNA structuromes in HIV, yeast, Arabidopsis, and mammalian cells and tissues have revealed the regulatory effects of RNA structure on messenger RNA (mRNA) polyadenylation, splicing, translation, and decay [21]. A recent study reported two-nucleotide (adenosine and cytosine)-based RNA structurome using a DMS-seq method and examined RNA structural flexibility during 2–6 h.p.f. in zebrafish embryogenesis. This study showed that RNA structure is shaped by the ribosome in a translation-dependent manner, and found dynamic 3′ UTR structures contain RNA-decay elements [30].

Here we investigated the RNA structure-based regulation of transcriptomic fate and function during zebrafish early embryogenesis. We generated the global maps of RNA structures at various stages during early embryogenesis in zebrafish, covering all four nucleotides of several thousands of transcripts. The results revealed the dynamic nature of RNA structuromes during zebrafish early development.

Comparative analysis of RNA structures at different time points revealed many hot regions of structural changes, which are enriched for *cis*-regulatory elements corresponding to specific RBPs. The previous studies [29, 30] also reported that the *cis*-regulatory elements were enriched in structural changes region in 3′ UTR by comparative analysis, but the intrinsic bias of DMS to A and C leads to the uncomplete identification of the structure-based *cis*-elements. With the all-4-base structural maps at six early developmental stages in zebrafish, we were therefore able to reconstruct large-scale structure-based posttranscriptional regulomes for zebrafish in different developmental contexts of early embryogenesis. Three factors are required for the regulome construction and the following desirable analyses: (1) the RNA structures of multiple developmental stages along the full zebrafish early embryogenesis were probed, which allow the identification of structurally variable regions along the development process; (2) by using icSHAPE, we obtained RNA structure maps of all four nucleotides enabling the motif search in those structurally variable regions; and (3) about 4000 transcripts were probed, which is also critical for the construction of a large-scale regulome.

From the analysis of the large-scale structure-based regulomes, we predicted and finally validated that the RBP Elavl1a regulates zebrafish maternal RNA stability in a structure-switch fashion, i.e., Elavl1a stabilizes its bound maternal mRNAs, whereas the secondary structure formation in maternal decay RNAs during 4–6 h.p.f. period blocks Elavl1a’s binding and consequently leads to maternal RNA degradation. It is known that association of RNA molecules with RNA-binding proteins (RBPs) is crucial for RNA metabolism, such as splicing and degradation in eukaryotes. Several RBPs have been demonstrated to be involved in maternal gene posttranscriptional regulation in different organisms. In *Drosphila*, Pumilio participates in deadenylation, translation repression, and maternal mRNA clearance, and Smaug can initiate poly(A) tail shortening followed by mRNA elimination [3]. However, RNA sequence motifs are not sufficient for association with RBPs, and RNA secondary structure also plays a repressive role in RBP binding [46]. This is supported by our findings that RBP Elavl1a regulates zebrafish maternal RNA stability in an RNA structure-dependent fashion. RNA structures inside a living cell can be affected and undergo changes by many factors, such as temperature, ion strength, crowding effect, cellular energy state, and the binding of RBPs including ATP-dependent RNA helicases, chaperone proteins, and others [47]. As Elavl1a itself does not appear to modulate the RNA structure at the Elavl1a binding sites from our findings, it is likely that other cellular factors may be implicated in the process of RNA structural switch. Moreover, Elavl1a-deficiency blocks normal development of zebrafish. Thus, association of RBPs with variable RNA structure plays a critical role during zebrafish embryogenesis.

Overall, our study reveals the extensive role of RNA structures in regulating early embryogenesis in vertebrates. By generating the RNA structure landscape and structure-based regulomes, we can predict the on-and-off binding of RBPs on the transcriptome in early embryogenesis. Further investigations with additional complementary approaches, including experiments to perturb RNA structures in vivo, are warranted to fully dissect the complex posttranscriptional regulatory network and to facilitate functional studies on early embryogenesis in vertebrates.

## Methods

### Animal models

Zebrafish wild-type strain AB was raised in system water at 28.5 °C under standard conditions. The zebrafish embryos were acquired by natural spawning.

### Cell lines

293T cells were originally purchased from ATCC, authenticated by short tandem repeat (STR) analysis, and routinely confirmed to be free of mycoplasma. 293T cells were maintained in standard DMEM (Gibco) supplemented with 10% fetal bovine serum (Gibco) and 1 × penicillin/streptomycin (Invitrogen) in standard humidified 5% CO_2_, 37 °C cell culture incubator.

### Morpholinos, vector construction, mRNA synthesis, injection

Morpholino oligonucleotides (MOs) of genes include the following: *elavl1a* atgMO: 5′-TGTGGTCTTCGTAACCGTTCGACAT-3′, Control MO: 5′-CCTCTTACCTCAGTTACAATTTATA-3′. MOs were purchased from GeneTools. MOs (6 ng for *elavl1a*, 6 ng for Control MO) were injected into one-cell stage embryos. For *elavl1a* overexpression experiments, *myc*-tagged or *flag*-tagged *elavl1a* was cloned into pCS2+ vector, mRNAs were generated using mMACHINE™ (Invitrogen) and injected into one-cell stage embryos. For mRNA structure reporter experiments, GFP reporters and Control mCherry were subcloned into pCS2+ vectors, capped and polyadenylated mRNAs were generated using mMACHINE™ and Poly(A) tailing kit (Invitrogen) according to the manufacturer’s protocol. A total of 20 pg of either GFP reporter mRNA and 20 pg of mCherry mRNA were injected into one-cell stage embryos.

### Generation of mutant by CRISPR/Cas9

The *elavl1a* mutant was generated using CRISPR/Cas9, and the method for Cas9 mRNA and guide RNA synthesis was described previously [48]. pXT7-Cas9 was used for Cas9 mRNA transcription by T7 mMessage Machine kit (Invitrogen, AM1344). Cas9 mRNA was purified using RNA clean Kit (TIANGEN, DP412). The *elavl1a* gRNA, target sequence: GGCCAAGCTCTGACTCCATCAAGG, was generated with in vitro transcription by T7 RNA polymerase (Promega, P2075). Mutant identification was carried out by DNA sequencing analysis.

### Microscopy

Bright-field and fluorescent images of embryos were observed with a Nikon SMZ1500 fluorescent microscope and captured with a Nikon digital camera. The relative mean fluorescence intensities were analyzed using ImageJ.

### Whole-mount in situ hybridization

Whole-mount in situ hybridization was carried out using a ZF-A4 in situ hybridization machine (Zfand, China) with digoxigenin-uridine-5′-triphosphate (Roche) labeled single-stranded RNA probes for *elavl1a*. For probe synthesis, PCR products of *elavl1a* F:5^′^-ATGCCAGGCCAAGCTCTGACTC-3^′^, *elavl1a* R:5^′^-TGCAGGCAAACCCTGCTTTCTAC-3^′^ were cloned into pGEM-T vector (Promega), and the RNA probe was transcribed with T7 RNA polymerase (Promega). After hybridization, RNA probes were detected by AP-conjugated anti-DIG antibody (Roche), and color reactions were carried out using BM purple (Roche) as substrate.

### Western blotting

Western blotting was performed as previously reported [49] using the following antibodies: monoclonal anti-Hur (Invitrogen, 39–0600), anti-β-actin antibody (Cell Signaling Technology, 4967), rabbit anti-Flag-HRP (Sigma-Aldrich, F7425).

### Manual SHAPE analysis

The manual SHAPE analysis assay was performed as a previously reported method [19]. RNA probes were synthesized by the GenScript Biotech Corp (5′-GGCCUUCGGGCCAAUCGAUCCGGUUCGCCGGAUCCAAAUCGGGCUUCGGUCCGGUUC-3′). RNA was heated in metal-free water for 2 min at 95 °C. The RNA was then flash-cooled on ice. The RNA 3X SHAPE buffer (333 mM HEPES, pH 8.0, 20 mM MgCl_2_, 333 mM NaCl) was added and the RNA was allowed to equilibrate at 37 °C for 10 min. Then, injecting the folded RNA probe to each stage (egg, 1-cell, 4-cell, 64-cell, sphere, shield) of zebrafish embryos (10 ng/embryo). After injection, NAI-N_3_ modification or vehicle control DMSO (unmodified group) was performed immediately. Total RNA was isolated by using TRIzol® Reagent (Invitrogen, 15596018). Cy3-labeled DNA primer (5′-GAACCGGACCGAAGCCCG-3′) was annealed to 20 μg of total RNA by incubating at 95 °C for 2 min followed by a step-down cooling (2 °C per s) to 4 °C. To the reaction first-strand buffer, dithiothreitol (DTT) and dNTPs were added. The reaction was pre-incubated at 52 °C for 1 min, then SuperscriptIII (2 U μl^− 1^ final concentration) was added. Extensions were performed for 10 min. To the reaction, 1 μl of 4 M NaOH was added and allowed to react for 5 min at 95 °C. Ten microliters of Gel Loading Buffer II (Ambion) was then added, and cDNA extensions were resolved on 8% denaturing (7 M urea) polyacrylamide gels (29:1 acrylamide: bisacrylamide, 1 × TBE). cDNA extensions were scanned on a Typhoon 9400 (GE Healthcare, USA) imager.

### In vivo SHAPE modification

In vivo SHAPE modification of zebrafish RNAs was performed as described previously [19] with some modifications. Briefly, 500 zebrafish embryos for each stage or condition were rinsed once and collected into a 15-ml tube in 2.5 ml fish water (6 mM NaCl, 0.6 mM KCl, 0.2 mM NaHCO_3_, 0.9 mM CaCl_2_). In total, 500 μl of 2 M NAI-N_3_ in DMSO (modified group) or vehicle control DMSO (unmodified group) was added drop-wise, immediately mixed by inversion, and incubated at 28.5 °C on end-over-end rotation for 5 min. Total RNA was isolated by using TRIzol® Reagent (Invitrogen, 15596018). mRNA was extracted with using Dynabeads® mRNA Purification Kit (Ambion, 61006).

### icSHAPE deep-sequencing library preparation

The purified mRNA was subjected to icSHAPE library preparation as previously described with some modifications [19]. The in vivo modified RNA and unmodified RNA were biotinylated by copper-free click reaction, followed by fragmentation. The fragmented RNA was end repaired by incubation at 37 °C for 1 h with the following mix (70 mM Tris 7.0, 18 mM MgCl_2_, 5 mM DTT, 4 U/μl RiboLock, 0.1 U/μl FastAP (Life Technology), 2 U/μl T4 PNK (NEB)) before 3′ adapter ligation with addition of 10 μl ligation mix (5 mM DTT, 0.5 μM 5′ adenylated and 3′-blocked linker (3′-bio for unmodified, 3′-ddc for modified), 0.66 U/μl T4 RNA ligase (NEB, M0437M), 15% PEG8000, 1X RNA ligase buffer) and incubation at 25 °C for extra 3 h. Then the excess adaptor was removed as described below. The purified RNA was incubated in the mix of 1 μl FastAP, 0.2 μl SSB (Promega), 0.8 μl Ribolock, and 1 μl 5′ Deadenylase (NEB) in 1× NEB buffer 2 (NEB) at 30 °C for 90 min. And then 1 μl RecJf (NEB) was added with another incubation at 37 °C for 1 h. The following procedures, including reverse-transcription, biotin-streptavadin enrichment, size selection of cDNA, circularization, and PCR amplification, were the same as described in the standard protocol.

### Elavl1a iCLIP

iCLIP was carried out as previously described [50]. *flag-elavl1a* mRNA-injected embryos were collected at 4 h.p.f. and 6 h.p.f.. A total of 400 zebrafish embryos were irradiated twice with 0.8 J/cm^2^ (Stratalinker 2400, Stratagene), lysed, and subjected to mild RNA fragmentation. Crosslinked RNA protein complexes were immunopurified using Anti-FLAG M2 Magnetic Beads (Merck, M8823) for 4 h.p.f. and 6 h.p.f. at 4 °C. RNA was extracted and subjected to library construction using Smarter smRNA-Seq kit (Clontech Laboratories Inc).

### RNA-seq

Total RNA was isolated from zebrafish embryos at different time points with TRIzol reagent and mRNA was further purified using Dynabeads mRNA purification kit (Ambion, 61006). Fragmented mRNA was used for library construction using the KAPA Stranded mRNA-Seq Kit (KAPA, K8401) according to the manufacturer’s protocol.

### Elavl1a RIP

*myc-elavl1a* mRNA-injected embryos were collected at 4 h.p.f. and 6 h.p.f., further lysed in NETN lysis buffer (150 mM NaCl, 0.5% NP-40, 50 mM Tris-HCl, pH 7.4). The Elavl1a RIP was carried out with a modified procedure [49]. In brief, lysate was incubated with Anti-Myc Agarose Affinity Gel antibody produced in rabbit (Sigma-Aldrich) for 4 h at 4 °C. After washing, proteins were digested by 4 μg μl^− 1^ proteinase K (Roche, 03115828001) in 200 μl PK buffer (50 mM NaCl, 100 mM Tris-HCl pH 7.4, 10 mM EDTA) for 20 min at 37 °C, followed by incubation with 200 μl PK-urea buffer (100 mM Tris-HCl pH 7.4, 50 mM NaCl, 10 mM EDTA, 7 M urea) for 20 min at 37 °C. After washing, RNA was collected by EtOH precipitation and then used for RT-qPCR.

### In vivo isolation of mRBPs from zebrafish embryos

The zebrafish mRBP capture was performed as previously described [50]. Briefly, 1000 zebrafish embryos at 0 h.p.f.(fertilized egg), 0.4 h.p.f.(1-cell), and 4 h.p.f. (sphere) were irradiated twice with 0.8 J/cm^2^ UV (254 nm) and snap frozen in liquid nitrogen (UV+), while nonirradiated embryos were served as negative controls (UV−). The embryos were subsequently lysed in lysis/binding buffer (100 mM Tris-HCl, pH 7.5, 10 mM EDTA pH 8.0, 500 mM LiCl, 1% [w/v] lithium-dodecyl sulfate (LiDS), 5 mM DTT, Protease Inhibitor Cocktail) and subjected to two rounds of 1 h mRNA capture at room temperature using 200 μl Dynabeads™ Oligo (dT)_25_ (Invitrogen). After three times of 10 min washes with Lysis/binding buffer, beads were washed three times with NP40 wash buffer (50 mM Tris-HCl, pH 7.5, 140 mM LiCl, 2 mM EDTA pH 8.0, 0.5% NP40, 0.5 mM DTT). mRNPs were heat eluted from the beads in 20 μl low-salt buffer (10 mM Tris-HCl, pH 7.5) at 70 °C. Proteins were released from mRNPs by 1 h RNase I (Ambion) treatment at 37 °C in low salt buffer and separated on a 4–12% NuPAGE NOVEX gradient gel in NuPAGE buffer (Thermo). The protein-containing gel was analyzed by mass spectrometry in the Institute of Biophysics, Chinese Academy of Science. The data files have been uploaded to http://www.peptideatlas.org with the access number: PASS01264.

### Protein purification in mammalian cells

293T cells were transiently transfected with pCS2-Flag-Elavl1a plasmids using PEI transfection reagent. Forty-eight hours later, cells were lysed with lysis buffer (50 mM Tris-HCl, pH 7.4, 500 mM NaCl, 1% NP-40, 1× Protease inhibitor cocktail) and then sonicated (10% output, 10 s pulse-on, 20 s pulse-off) for 1 min by a Sonic Dismembrator (Thermo Fisher). After removing the cell debris through centrifugation at 13,300 rpm for 20 min, the lysates were incubated with anti-Flag M2 Affinity Gel (Sigma-Aldrich) for 4 h at 4 °C. After washing with lysis buffer for five times and TBS buffer (20 mM Tris-HCl pH 7.4, 150 mM NaCl) for twice, the bead-bound proteins were eluted with 1 mg/ml 3× Flag peptide (Sigma-Aldrich) for 1 h at 4 °C. The elute containing purified protein was condensed using VIVASPIN 500 (Sartorius Stedim Biotech) and quantified by coomassie brilliant blue staining and western blotting.

### In vivo RNA pulldown assay

The biotin-labeled RNA probes were synthesized by the GenScript Biotech Corp. In vivo RNA pulldown assays were carried out using zebrafish embryo extracts as previously described [51] with some modifications. RNA was heated in metal-free water for 2 min at 95 °C. The RNA was then flash-cooled on ice. The RNA 3X SHAPE buffer (333 mM HEPES, pH 8.0, 20 mM MgCl_2_, 333 mM NaCl) was added, and the RNA was allowed to equilibrate at 37 °C for 10 min. Zebrafish embryo extracts were precleared for 1 h at 4 °C by incubation with streptavidin-conjugated magnetic beads (NEB) in binding buffer (50 mM Tris-HCl pH 7.5, 250 mM NaCl, 0.4 mM EDTA, 0.1% NP-40, 1 mM DTT) supplemented with 0.4 U/μl RNasin (Promega). Biotin-labeled RNA oligonucleotides were incubated with precleared nuclear extracts for 2 h at 4 °C under gentle rotation together with streptavidin-conjugated magnetic beads which were precleared by incubation with 0.2 mg/ml tRNA (Sigma) and 0.2 mg/ml BSA (Amresco) for 1 h at 4 °C under gentle rotation. Beads were washed three times with wash buffer (50 mM Tris-HCl pH 7.5, 250 mM NaCl, 0.4 mM EDTA, 0.1% NP-40, 1 mM DTT, 0.4 U/μl RNasin (Promega)). For western blotting analysis, samples were separated on SDS-PAGE and transferred onto PVDF membrane. After blocking with 5% non-fat milk in TBST for 1 h, the membrane was then incubated for 1 h at 4 °C with Monoclonal anti-Hur (Invitrogen, 39–0600) diluted at 1:1000 in 5% milk. Protein levels were visualized using ECL Western Blotting Detection Kit (GE Healthcare).

### In vitro RNA pulldown assay

In vitro RNA pulldown assay was performed according to the previously reported method [51] with some modifications. Generally, RNA was heated in metal-free water for 2 min at 95 °C. The RNA was then flash-cooled on ice. The RNA 3X SHAPE buffer (333 mM HEPES, pH 8.0, 20 mM MgCl_2_, 333 mM NaCl) was added and the RNA was allowed to equilibrate at 37 °C for 10 min. Then, 10 pmol of purified Flag-Elavl1a protein and 10 pmol of biotin-labeled probes were incubated with 15 μl streptavidin-conjugated magnetic beads (NEB) in binding buffer (50 mM Tris-HCl pH 7.5, 250 mM NaCl, 0.4 mM EDTA, 0.1% NP-40, 1 mM DTT, 0.4 U/μl RNase inhibitor) for 1 h at 4 °C. After washing with binding buffer for three times, the bead-bound proteins were heated in NuPAGE™ LDS Sample Buffer (4×) (Invitrogen) and then separated on the NuPAGE™ 4–12% Bis-Tris Gel (Invitrogen), and subjected to western blotting analysis with anti-Flag antibody (Sigma-Aldrich, F7425).

### Electrophoretic mobility shift assay (EMSA)

Purified Flag-tagged Elavl1a proteins were diluted to a series of concentrations of 0.2 μM, 0.5 μM, 1 μM, and 2 μM in binding buffer (50 mM Tris-HCl pH 7.5, 100 mM NaCl, 0.4 mM EDTA, 0.1% NP-40, and 40 U/ml RNasin, 1 mM DTT, 50% glycerol, 5 ng/μl BSA). One microliter synthesized Cy3-labeled RNA probes (100 nM final concentration) and 1 μl purified protein (10 nM, 50 nM, 100 nM, and 200 nM final concentration, respectively) were mixed and incubated at room temperature for 30 min. Then, 1 μl glutaraldehyde (0.2% final concentration) was added into the mixture which was incubated at room temperature for 15 min. The entire 11 μl RNA protein mixture was mixed with 5 μl 5× Hi-Density TBE Sample buffer and separated on 6% TBE gel on ice for 30 min at 80 V. The gel was scanned on a Typhoon 9400 (GE Healthcare, USA) imager. Quantification of each band was carried out using ImageJ. The RNA binding ratio at each protein concentration was determined by (RNA protein)/((free RNA) + (RNA protein)).

### Quantitative reverse-transcription PCR

Quantitative reverse-transcription PCR (RT-qPCR) was carried out to examine the relative abundance of target RNA. 0.1 μg RNA were used for cDNA synthesis using RevertAidTM First Strand cDNA Synthesis Kit (Thermo). Experiments were performed with Takara SYBR Premix Ex Taq (Takara) according to the manufacturer’s instructions and examined by a CFX96 Real-Time PCR System (Bio-Rad). *P < 0.05; **P < 0.01; ***P < 0.001. The primers used for RT-qPCR in this study are listed in Additional file 10.

### icSHAPE reactivity calculation

#### Preprocessing

Raw sequence reads were first trimmed to remove 3′ adaptors by cutadapt (V 1.16), filtered out low-quality bases with Trimmomatic [52] (V 0.33), then collapsed to remove PCR duplicates and trimmed the leading 13-nt UMIs. Processed reads were mapped to Refseq zebrafish transcriptome (Z10) downloaded from UCSC Genome Browser by using bowtie2 with the parameters (--non-deterministic) recommended by icSHAPE.

#### Reactivity calculation

icSHAPE reactivity was calculated as previously described [19, 53] with some modifications. RT stop for each base was counted and replicates were combined. The combined RT stop for each base was normalized using a 200-nt sliding window with 30 nt per step. In each window, the RT stop was normalized with the average of the RT stop values rank from 90th percentile to 95th percentile then scaled up to 100 to give most of the RT stop values between 0 and 100. The final RT stop for each position was calculated as the average of the RT stop value for the certain position in the covered windows. Then the normalized RT stop was used to calculate icSHAPE reactivity for each base as previously described. We filtered out the transcripts with average RT stop < 2 (−T 2) and base density < 200 to get the final icSHAPE reactivity profile.

### Identification of structurally variable nucleotides and regions and “hot” structurally variable sites

For structurally variable nucleotides, we chose the same cutoff 0.2 used in our previous study [33] to distinguish a base with or without differential icSHAPE reactivity score. Additionally, we performed icSHAPE on folded spike-in RNAs. We found more than 98.5% bases with differential icSHAPE reactivity score of lower than 0.2 between replicates (Additional file 1: Fig. S2b). These results suggest that the cutoff of Δreactivity score > 0.2 is enough to filter out most of the technical noise and can be used to assess the significant structural change for a single nucleotide.

For structurally variable regions, we used the similar strategy to choose the cutoff by ranking all windows according to the average Δreactivity score. About 95% windows have the average Δreactivity score lower than 0.05. For every window, we also calculated the significance of the difference by using the two-sided paired Student’s *t*-test. *P* value < 0.05 was considered as a significant structural change. In sum, the average Δreactivity score > 0.05 and *P* value < 0.05 (two-sided paired Student’s *t* test) were used to assess the significant structural change for a 10-nt sliding window. The whole process is very similar as the differential gene expression analysis (Additional file 1: Fig. S2c). In order to identify structurally variable regions within individual transcript between two samples, icSHAPE reactivities per transcript were first subdivided into 10-nt disjoint windows. Only the windows with ≥ 80% bases of valid icSHAPE reactivity in both comparing samples were kept for the further analysis. The windows with difference of average icSHAPE reactivity > 0.05 and *P* value < 0.05 (two-sided paired Student’s *t* test) were marked as less structural windows; < − 0.05 and *P* value < 0.05 were marked as more structural windows; and others were marked as stable ones. The less structural and more structural windows were used to define structurally variable regions. Next, we extended these windows with 10-nt flanking regions and then merged the overlapped extended windows and divided them into 30 nt regions to remove redundancy. These re-bounded windows were finally defined as variable structure regions with the same annotation as that of their origin.

For the definition of “hot” structurally variable sites, the structurally variable regions that are shared by two comparisons, e.g., comparison of 0 h.p.f. and 0.4 h.p.f. stage and comparison of 1 h.p.f. and 2 h.p.f., are defined as two-way “hot” structurally variable regions. Those shared by three comparisons are termed three-way “hot” structurally variable regions and so on.

### Enrichment of structurally variable regions in different parts of transcripts

To assess the enrichment of the structurally variable windows in 5′ UTR, CDS, and 3′ UTR, we assigned each window to one of the three nonoverlapping transcript segments: 5′ untranslated region (UTR), coding sequence (CDS), and 3′ untranslated region (UTR) by applying BEDTools [54] (bedtools intersectBed –f 0.51). Take the relative length ratio (5′ UTR:CDS:3′ UTR = 150:1250:460) of each segment occupied in the zebrafish transcriptome into consideration, which is estimated by comparing the average length of the three fragments of the transcripts, the enrichment ratio of each segment at certain stage was defined by using the raw counts divided by expected counts assuming that the structurally variable regions uniformly distributed across the transcripts. Fisher’ exact test was performed to test the significance of difference between raw counts and expected counts.

### De novo motif discovery and enrichment analysis of structurally variable regions

To identify sequence or RBP binding motifs enriched in the structurally variable regions, we performed two kinds of analyses.

#### De novo motif discovery in structurally variable regions

To explore the sequence feature of structurally variable windows at 3′ UTR, we used MEME suite Dreme [55], a tool for short motif discovery, to identify potential sequence motifs. First, we generated the corresponding shuffled control regions on the same transcript using bedtools shuffleBed. Next, Dreme (-norc-mink 5 -rna) was applied to search motif whose length is no less than 5 nucleotides. To identify regulatory sequence motifs that are similar to the enriched sequence motifs, TOMTOM [56] (-min-overlap 5 -dist pearson-evalue -thresh 5 -norc) was used to compare the enriched motifs with known regulatory sequence motifs. Here we collected a known reference motif set, including 55 conservative RBPs from CISBP-RNA Database [57] (http://cisbp-rna.ccbr.utoronto.ca/), and ELAVL1 from ATtRACT database [58] (https://attract.cnic.es/).

#### RBP binding motif enrichment analysis

To determine whether specific RBP binding motifs are enriched in structure regions and identify the potential binding sites, FIMO [59] tool in the MEME suite was used to search the occurrences of the motifs in the variable regions at 3′ UTR or reference set with the parameter “--thresh 0.001.” The reference set was generated by randomly selection of regions of the same length 30 nt from the same set of transcripts containing the structurally variable regions of interest with bedtools shuffleBed but excluding the variable regions. For each motif separately, Fisher’s exact test was performed to estimate the enrichment ratio which was defined as (a/b)/(c/d), a: number of sequences with significant FIMO hits in the dynamic set, b: number of sequences with no significant hits in the dynamic set, c: number of sequences with significant FIMO hits in the reference set, and d: number of sequences with no significant hits in the reference set. The motifs with false discovery rate adjusted *P* values < 0.05 were considered as significantly enriched.

### Data processing and analysis of RNA-seq

The quality of raw sequencing reads was screened using FastQC (http://www.bioinformatics.babraham.ac.uk/projects/fastqc/), and low-quality bases were trimmed and filtered by cutadapt (V 1.16) and Trimmomatic [52] (V 0.33). Processed reads were mapped to zebrafish transcriptome (Z10) from Refseq annotation using bowtie2 [60] (V 2.2.9) with default parameters. After mapping quality (≥ 20) filtering using SAMtools [61] (V1.0), read counts and corresponding RPKM were calculated by Perl script. The final RPKM of each transcript is the average of RPKMs in replicates.

The fold change of expression level of transcripts between samples were calculated using DEGseq [62] (V 1.28.0) package. Based on comparison of expression level between 2 h.p.f. and 6 h.p.f., we defined three groups of genes: (i) genes with RPKM > 1 at 2 h.p.f., log_2_ (FC) > log_2_ (1.2), and FDR < 0.05 were defined as maternal decay genes (*N* = 6589); (ii) genes with RPKM > 1 at 2 h.p.f., log_2_ (FC) < log_2_ (1.2), and FDR < 0.05 were defined as maternal stable genes (*N* = 2479); (iii) genes with RPKM < 1 at 2 h.p.f. and RPKM > 1 at 6 h.p.f. were defined as zygotic genes (*N* = 1225).

### Data processing and peak calling of iCLIP

#### Preprocessing and peak calling

The processing of iCLIP data is similar to pipeline as described in CTK tool (https://zhanglab.c2b2.columbia.edu/index.php/ICLIP_data_analysis_using_CTK). Firstly, the reads were trimmed off adapter with cutadapt followed by collapsing duplicates with CTK tool (fastq2collapse.pl). After stripping the 5′ degenerated barcode, we mapped the clean reads to the transcriptome using bwa (v0.7.17). For each time point (4 h.p.f. and 6 h.p.f.), two replicates were merged before subsequent peak calling. We used CTK tool CITS mode to call the peaks. The reads were clustered using tag2cluster.pl with parameters -big -s -maxgap “-1” and the peaks are identified using tag2peak.pl with parameters -big -ss -v --prefix “CITS” -gap 25 -p 0.05.

#### Binding motif identification

To identify the motif regions from iCLIP data, we first extended 20 nucleotides long from the truncation site to flanking regions. Only those sites annotated within 3′ UTR regions are retained. Then, we used MEME suite Dreme [55] to identify sequence motif in Elavl1a binding regions with minimum motif length of 7 (-k 7). The background regions were randomly selected on the same transcript using bedtools shuffleBed.

#### Meta-analysis of icSHAPE profile on Elavl1a binding sites

We calculated the metagene icSHAPE profile around the Elavl1a binding sites and the unbind control by averaging all valid icSHAPE reactivity upstream and downstream 20 nt of the center of the Elavl1a binding motif. The unbind control was a set of regions on the same set of transcripts containing the same sequence motif, excluding the Elavl1a binding sites.

### Data processing and analysis of mRBPs capture

For the mRBP capture data, LFQ intensity values of +UV (positive) and −UV (negative) sample are obtained from the detector with replicates (Additional file 3). For mRBP binding affinity at 4 h.p.f. stage, we directly compared the positive signal over negative signal intensity of the first two replicates to calculate the enrich folds, and two-sided unpaired Student’s *t* test was used to test the significance. Statistical significance was set to *P* value ≤ 0.05.

To more reliably compare the mRBPs binding across stages, we included additional two replicates and calculated the fold change of averaged positive signal over negative signal of all replicates as indicator of mRBPs binding.

### Gene ontology analysis

Gene ontology (GO) analysis was performed using the DAVID [63] website (https://david.ncifcrf.gov/). GO classification for biological process was performed using the genes with RPKM > 1 for respective zebrafish embryo developmental stage as background.

### Analysis of the icSHAPE reactivity at zebrafish RBP binding sites

The dataset of RBP binding sites of 23 iCLIPs in zebrafish embryos at 4 h.p.f. (sphere) stage was collected [32]. The binding sites of the 23 RBPs were extracted and merged. The whole transcript background were generated by shuffling on the same set of transcripts using bedtools shuffleBed. The RBP binding sites and shuffled background were kept for analysis if the percentage of the bases with valid icSHAPE reactivity is larger than 60%.

### Quantification and statistical analysis

Statistical comparison of structurally variable regions between stages was carried out with two-sided paired Student’s *t* test using t.test function in stats R package. Statistical significance was set to *P* < 0.05.

All the correlations and *P* values in Additional file 1: Fig. S1d, S2a, S3d, S5d were calculated by python package scipy.stats.pearsonr. Additional file 1: Fig. S1c used scipy.stats.spearman.

Statistical significance of the enrichment of adenosine and uracil in structurally variable bases was analyzed with Fisher’s exact test. *P* values were less than 2.23 × 10^− 308^ (Figs. 1b and 2b).

Statistical significance in differences of the averaged icSHAPE reactivity of transcripts between stages were analyzed with two-sided paired Student’s *t* test. Exact *P* values are listed in the figure (Fig. 1d).

Statistical significance in differences of icSHAPE reactivity between double-stranded and single-stranded nucleotides on conserved secondary structures in the 5′ UTR of *dgcr8* was analyzed with two-sided unpaired Student’s *t* test: single-stranded nucleotide, *n* = 29; double-stranded nucleotides, *n* = 62. *P* value is listed in the figure (Additional file 1 Fig. S1f).

Statistical significance in differences of the pulldown mRBP abundance between stages was analyzed with two-sided Kolmogorov-Smirnov test. Exact *P* values are listed in the legend (Additional file 1 Fig. S1i).

Statistical significance of the enrichment of the structurally variable regions in 3′ UTR was analyzed with Fisher’s exact test. Statistical significance was set to *P* < 0.05. *P* values for each test were all less than 2.23 × 10^− 308^ (Fig. 2e).

Statistical significance of the enrichment of the motifs in structurally variable regions was analyzed with Fisher’s exact test. Statistical significance was set to adjusted *P* < 0.05 (Fig. 2g, Additional file 1: Fig. S2g and Fig. 3a). Exact *P* values are listed in Additional file 5.

Statistical significance of the difference of the western quantification was analyzed with two-tailed unpaired Student’s *t* test (*n* = 3); *, *P* < 0.05; **, *P* < 0.01; ***, *P* < 0.001 (Fig. 3f, g, i, and j).

Statistical significance in difference of stability during 4 h.p.f. to 6 h.p.f. between groups was analyzed with two-sided Wilcoxon test using function wilcox.test in stats R package. Exact *P* values are labeled in the figure (Fig. 4b).

Statistical significance in differences of the fold change was carried out with two-tailed unpaired Student’s *t* test (*n* = 3); *, *P* < 0.05; **, *P* < 0.01; ***, *P* < 0.001 (Additional file 1 Fig. S4b).

Statistical significances in differences of average icSHAPE reactivity of Elavl1a binding sites between 4 h.p.f. and 6 h.p.f. were calculated by two-sided unpaired Student’s *t* test. Exact *P* values are labeled in the figure (Additional file 1 Fig. S4f).

Statistical significance of the comparison of the stability between groups was analyzed with two-sided Kolmogorov-Smirnov test. Exact *P* values are listed in figure and legend (Fig. 5c, d).

Statistical significance in differences of the expression estimated by qPCR was analyzed with two-tailed unpaired Student’s *t* test (*n* = 3); *, *P* < 0.05; **, *P* < 0.01; ***, *P* < 0.001 (Fig. 6c and Additional file 1: Fig. S6a).

Statistical significance in differences of the fluorescence intensity was analyzed with two-tailed unpaired Student’s *t* test (*n* = 3); *, *P* < 0.05; **, *P* < 0.01; ***, *P* < 0. 001 (Fig. 6d and Additional file 1: Fig. S6b).

All the statistical tests listed above, if not specified, were carried out with scipy Python package (https://www.scipy.org/) using functions: scipy.stats.mannwhitneyu, scipy.stats.ttest_ind, scipy.stats.ks_2samp, scipy.stats.fisher_exact.

## Supplementary information

Additional file 1: Supplementary Figures S1-S7.

Additional file 2: **Table S1.** Summary and statistics of icSHAPE, RNA-seq and iCLIP.

Additional file 3: **Table S2.** LFQ intensity of proteins in UV+ and UV- samples at 0, 0.4 and 4 h.p.f.

Additional file 4: **Table S3.** Structurally variable regions between neighboring stages and hot structurally variable regions.

Additional file 5: **Table S4.** Results of motif enrichment analysis in 3′ UTR structurally variable regions.

Additional file 6: **Table S5.** Summary of DLE element in structurally variable regions during early development and its associated biological function.

Additional file 7: **Table S6.** Elavl1a binding sites at 4 h.p.f. Elavl1a binding sites identified by Flag-Elavl1a iCLIP at 4 h.p.f. and 6 h.p.f.

Additional file 8: **Table S7.** Maternal and zygotic gene sets categorized by gene expression and SNP.

Additional file 9: **Table S8.** GO term enrichment analysis of down-regulated genes upon elavl1a knockdown at 6 h.p.f.

Additional file 10: **Table S9.** List of oligos used for this Study.

Additional file 11: Review history.
