# Supplementary material for: RNA structural dynamics regulate early embryogenesis through controlling transcriptome fate and function
Source: Genome Biol. 2020 May 18;21:120. doi: 10.1186/s13059-020-02022-2 (PMC7236375; doi:10.1186/s13059-020-02022-2)
Supplement: Supplementary file 1 — Supplementary Figures S1-S7. [file 13059_2020_2022_MOESM1_ESM.docx]

**Supplementary figures**


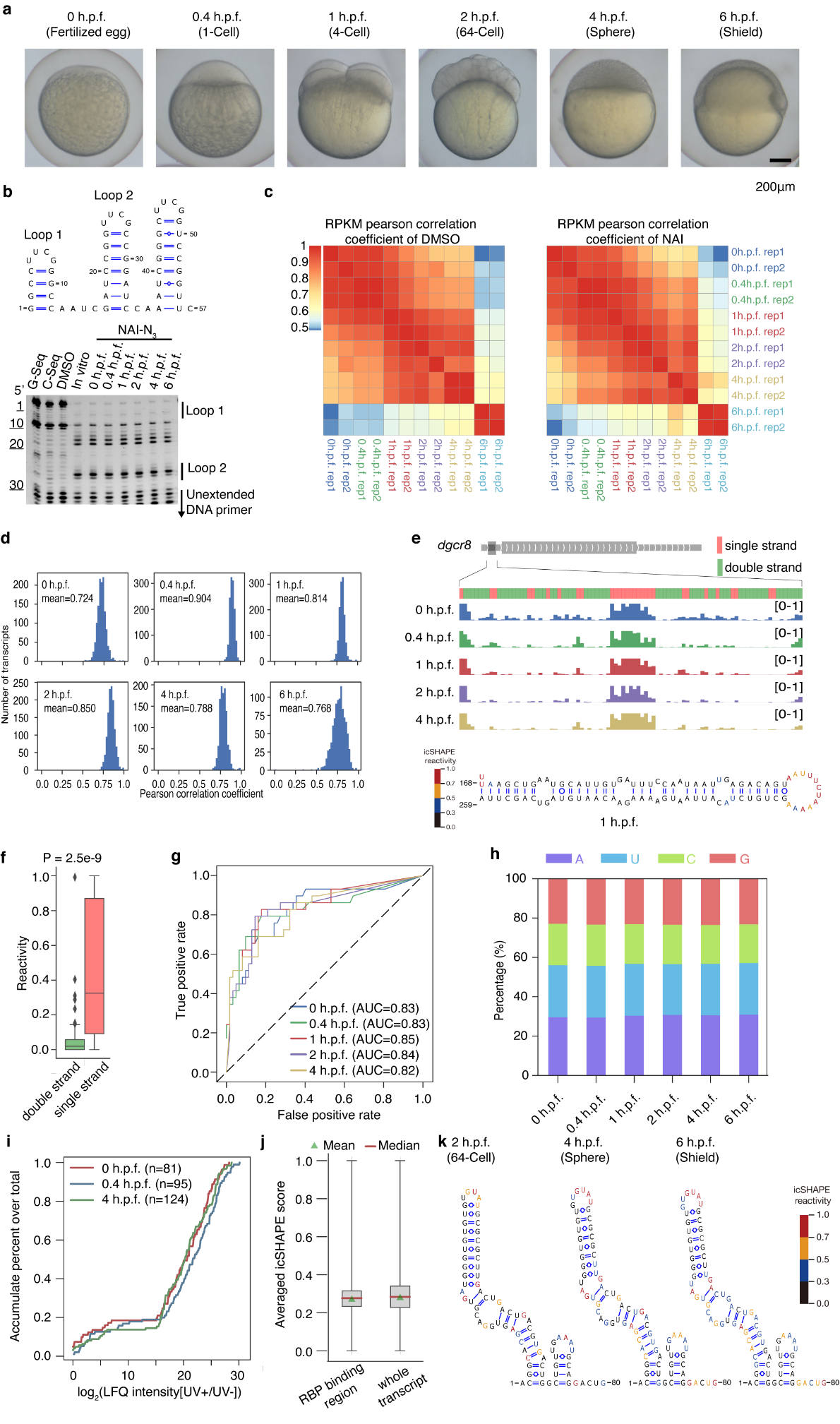


**Fig. S1 Validation of icSHAPE data quality.** (a) Zebrafish embryos at six different time points collected for icSHAPE profiling. (b) Denaturing gel analysis of folded RNA *in vivo* probed with NAI-N_3_ shows unbiased NAI-N_3_ probing at different time points. (c, d) Reproducibility of icSHAPE data across biological replicates at a per-transcript count level (c) and per-nucleotide rt-stop level (d) *in vivo* for each time point. (e) *In vivo* icSHAPE reactivity profile of the 5’ UTR of *dgcr8*. Accessibilities corresponding to annotated conserved secondary structures (gray rectangles) are highlighted (upper). The detailed icSHAPE reactivity and RNA structure model (1 h.p.f.) is also shown (lower). (f) Box plot comparing the distribution of *in vivo* icSHAPE reactivity (1 h.p.f.) and the pairing status of conserved secondary structures in the 5’ UTR of *dgcr8*. *P*-value was computed using two-sided unpaired Student’s t-test (single strand, n=29; double strand, n=62). (g) Receiver operating characteristic (ROC) curve for icSHAPE reactivity of each time point on conserved secondary structures in the 5’UTR of *dgcr8*. AUC, area under curve. (h) Nucleotide composition of profiled bases at each time point. (i) Cumulative distribution of the log2 fold of change of LFQ intensity of mRBPs between UV+ and UV- samples at 0, 0.4, 4 h.p.f. Statistical significances were calculated by the two-sided Kolmogorov-Smirnov test. The exact p-value is 0.21 for 0 h.p.f. vs 0.4 h.p.f., 0.17 for 0.4 h.p.f. vs 4 h.p.f., and 0.71 for 0 h.p.f. vs 4 h.p.f.. (j) Box plot shows very small differences between the icSHAPE reactivity scores at RBP binding sites and random positions on the whole transcripts in zebrafish. (k) RNA structure model of *kpna4* gene at 3’ UTR region at 2, 4, 6 h.p.f..

**
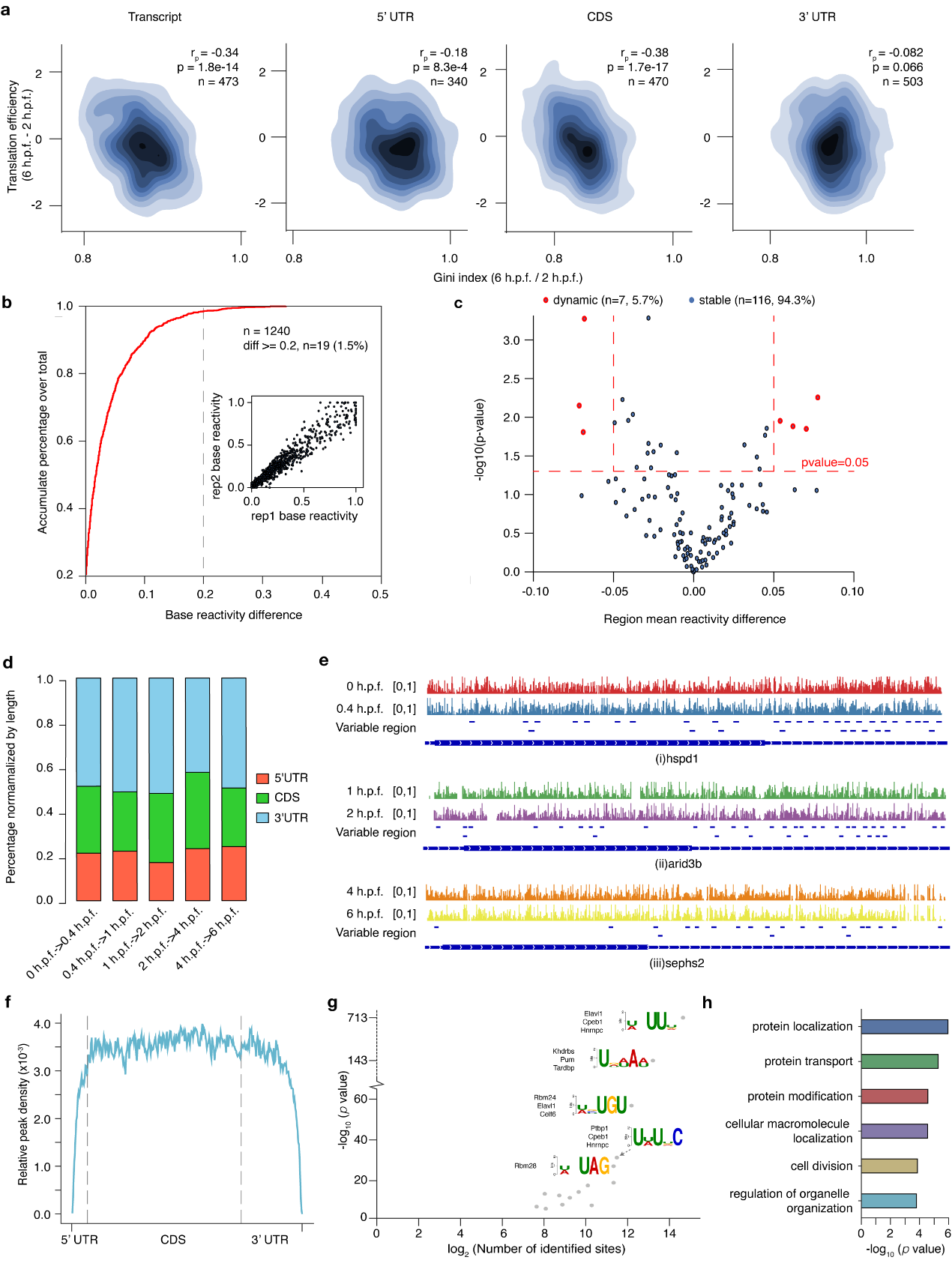
**

**Fig. S2 RNA structures are variable during zebrafish embryos development.** (a) Correlation between changes in translation efficiency and RNA accessibility (6/2 h.p.f.), for transcripts, 5′ UTR, CDS and 3′ UTR. Translation efficiency data was obtained from Subtelny et al., 2014. (b) Cumulative distribution of differential icSHAPE reactivity of spike-in RNAs between replicates at single nucleotide resolution. Inset scatter plot shows the icSHAPE reactivity of each nucleotide or 10 nt sliding window between replicates. (c) Volcano plot shows the distribution of differential averaged icSHAPE reactivity and log10 *P* values of 10nt sliding windows in spike-in RNAs between replicates. Statistical significant was calculated by two-sided paired Student’s t test. (d) Percentage of the normalized counts of structurally variable regions in each part of transcript in different comparisons. (e) Examples for genes with structurally variable region enriched in 3’UTR from different comparison. (ⅰ) *hspd1* between 0 h.p.f. and 0.4 h.p.f., (ⅱ) *arid3b* between 1 h.p.f. and 2 h.p.f., (ⅲ) *sephs2* between 4 h.p.f. and 6 h.p.f.. (f) Meta-gene profiling depicts the sub-transcript distribution pattern of 1-way structurally variable windows. (g) Scatter plot shows the significance and occurrences of *de novo* discovered sequence motif enriched in common structurally variable windows at 3’ UTR shared by at least two comparisons. *P* values were calculated by Fisher’s exact test. The RBPs with binding sites that are similar to the sequence motif are listed. (h) Gene set enrichment analysis of the transcripts that contain a DLE motif in structurally variable regions.


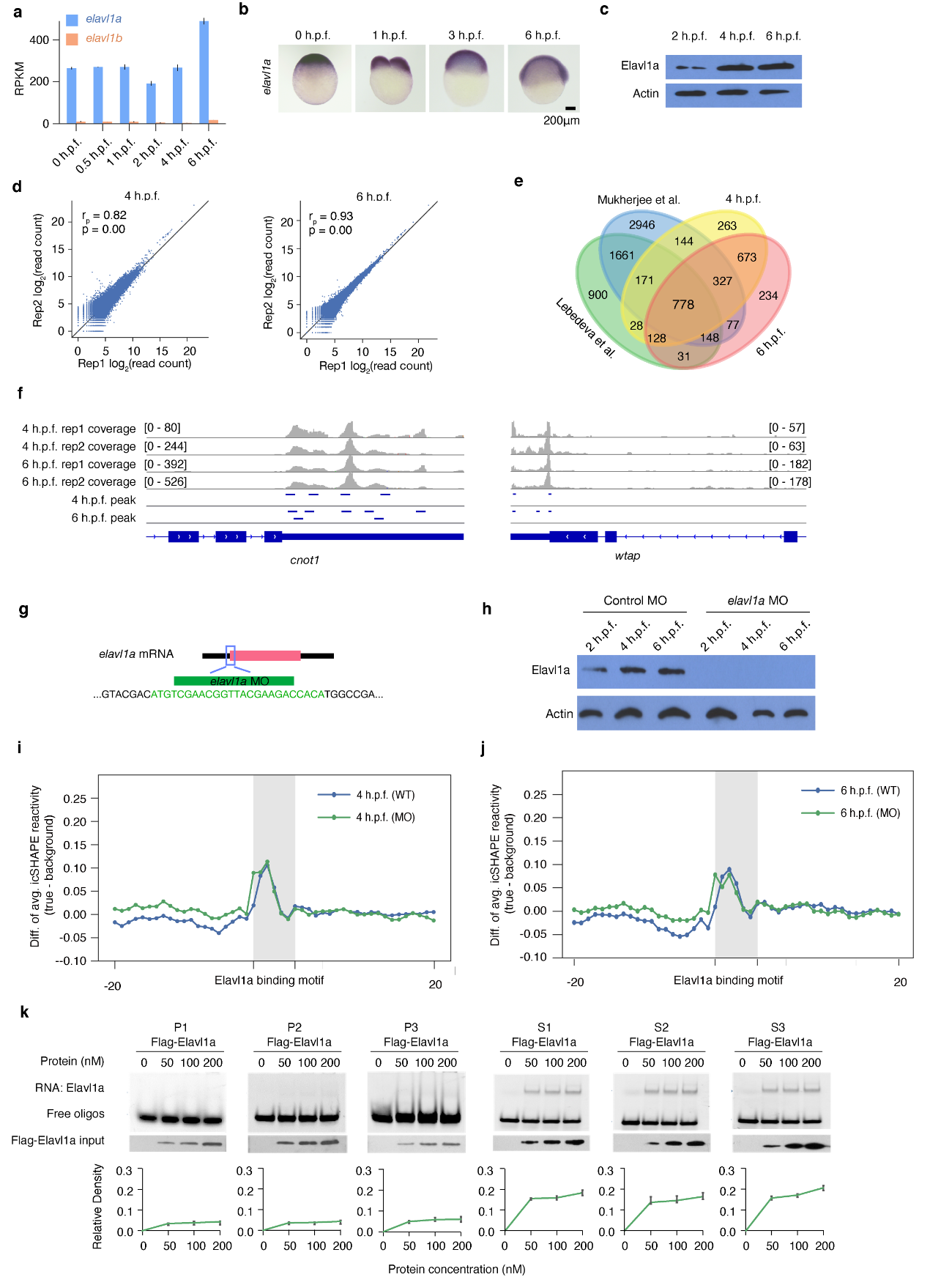


**Fig. S3. RNA structurally variable regions in 3’ UTRs are enriched with single-strand RBP Elavl1a.** (a) The mRNA expression level of *elavl1a* and *elavl1b* in zebrafish. (b) Whole-mount in situ hybridization (WISH) shows the *elavl1a* mRNA expression pattern in different developmental stages. (c) Western blotting shows the protein level of Elavl1a at 2 h.p.f., 4 h.p.f. and 6 h.p.f.. (d) RPKM correlation of iCLIP data between biological replicates. (e) Overlap of human ELAVL1 binding genes and zebrafish Elavl1a binding genes. Human ELAVL1 binding genes was obtained from Lebedeva et al., 2011 and Mukherjee et al., 2011. (f) IGV tracks displaying Elavl1a binding peaks. (g) Schematic of binding site of *elavl1a* morpholino (MO). (h) Western blotting verifies the effectiveness of *elavl1a* MO. (i-j) Averaged icSHAPE reactivity profiles in 4 h.p.f. (i) and 6 h.p.f. (j) between Elavl1a binding sites identified by iCLIP and non-binding sites with the same sequence motif, in the wildtype (WT) and Elavl1a deficiency (MO) embryos. (k) EMSA showing the binding ability of purified Flag-Elavl1a with six endogenous RNA probes containing Elavl1a binding sites. 100 nM of RNA probes was incubated with different concentrations of Flag-Elavl1a protein. Error bars, mean ± s.d., n = 3.

**
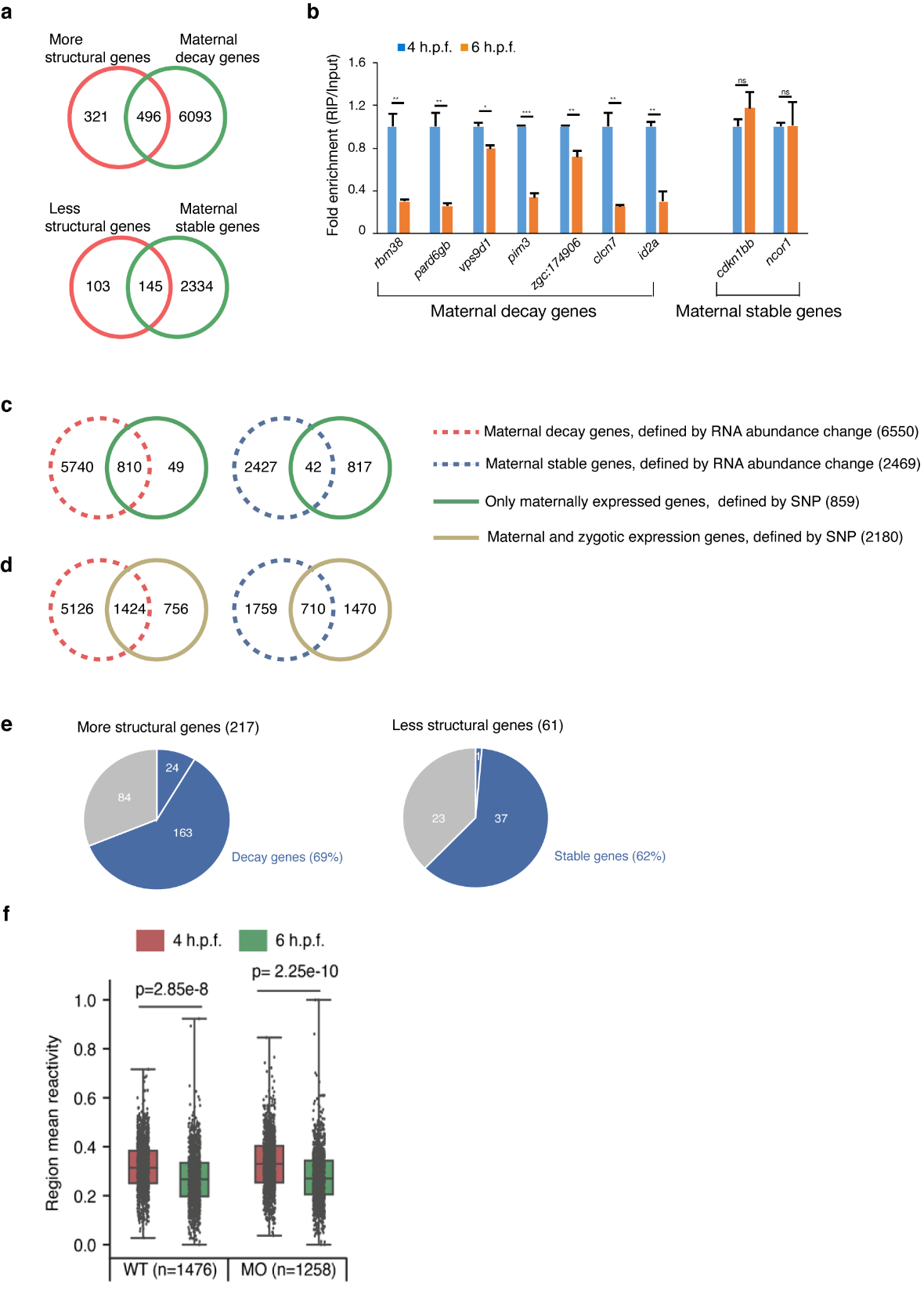
**

**Fig. S4 Maternal stable genes and maternal decay genes show differences in structure changes in Elavl1a binding regions.** (a) Overlap of maternal decay genes and transcripts with more structured Elavl1a binding sites, maternal stable genes and transcripts with less structured Elavl1a binding sites during the period of 4 h.p.f. to 6 h.p.f.. (b) RIP-qPCR shows the fold enrichment of transcripts with Elavl1a binding sites upon Elavl1a pull-down between 4 h.p.f. and 6 h.p.f.. Error bars, mean ± s.d., n = 3. *P* values were calculated using two-sided Student’s t-test. (c-d) Overlap of maternal decay and stable genes defined by only the change of RNA abundance and maternal-only (c) and maternal-and-zygotic (d) genes by using maternal vs. paternal SNPs. (e) Pie chart depicting the proportion of maternal decay genes (including both the maternal-only and the maternal-and-zygotic groups) in transcripts with more structural Elavl1a binding sites, and the proportion of maternal stable genes (both maternal-only and maternal-and-zygotic) in transcripts with less structural Elavl1a binding sites during the period of 4 h.p.f. to 6 h.p.f.. (f) Average icSHAPE reactivity scores around the Elavl1a binding sites that form more structure between 4 h.p.f. and 6 h.p.f., in WT and *elavl1a* MO samples. Statistical significances were calculated by the two-sided unpaired Student’s t test.

**
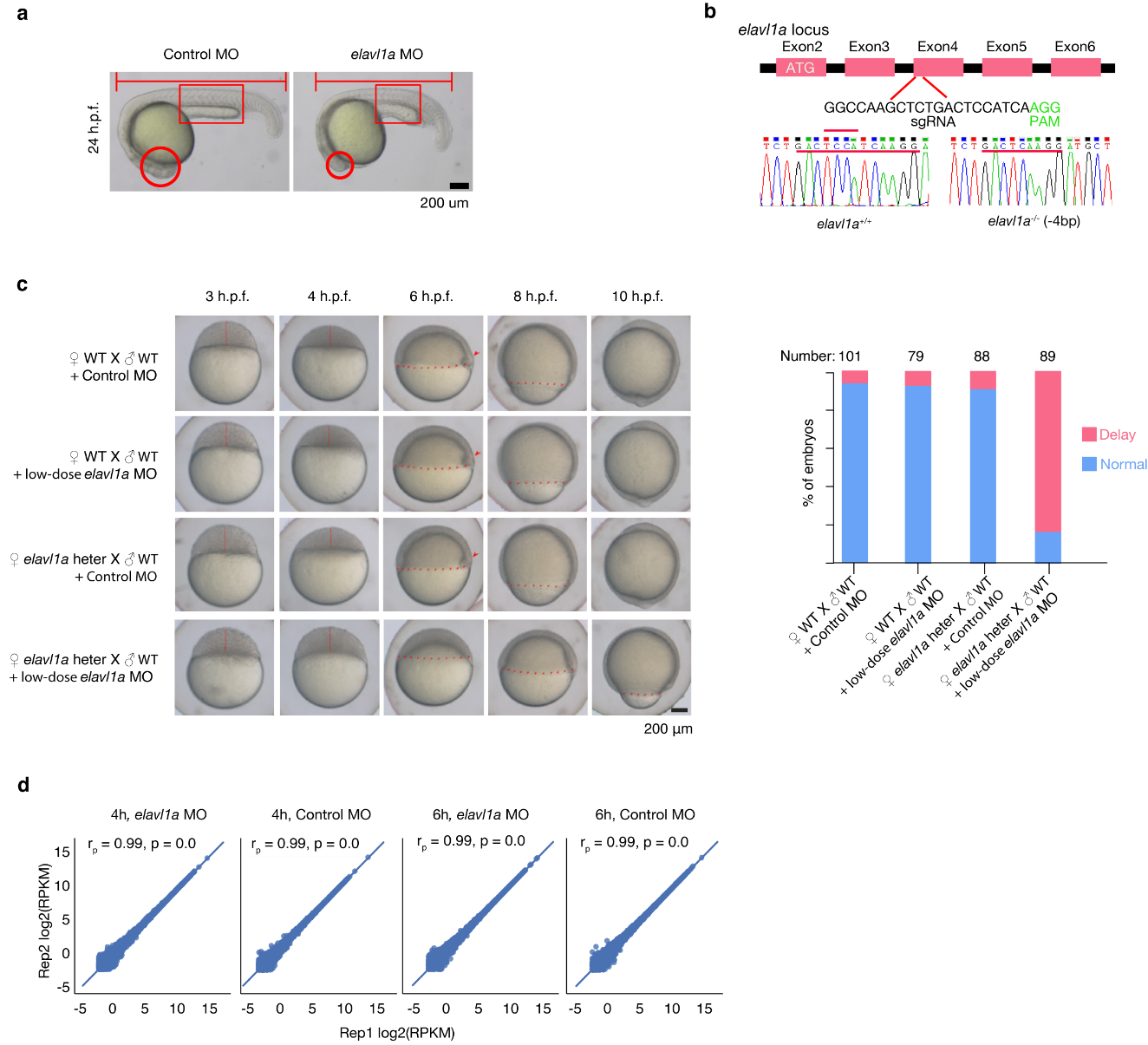
**

**Fig. S5 Elavl1a deficiency leads to developmental delay.** (a) Elavl1a deficiency leads to various malformations at 24 h.p.f.. (b) The upper panel showing the gRNA target site in the third exon of *elavl1a*. The lower panel showing the 4 bp deletion in the *elavl1a* mutants. (c) The embryos obtained from heterozygous females crossing with wild-type males, but not from wild-type mating, exhibited obvious developmental delay after low dose *elavl1a* MO injection. (d) RPKM Correlation of RNA-seq data between biological replicates for Elavl1a knockdown and control embryos at 4 h.p.f. and 6 h.p.f..

**
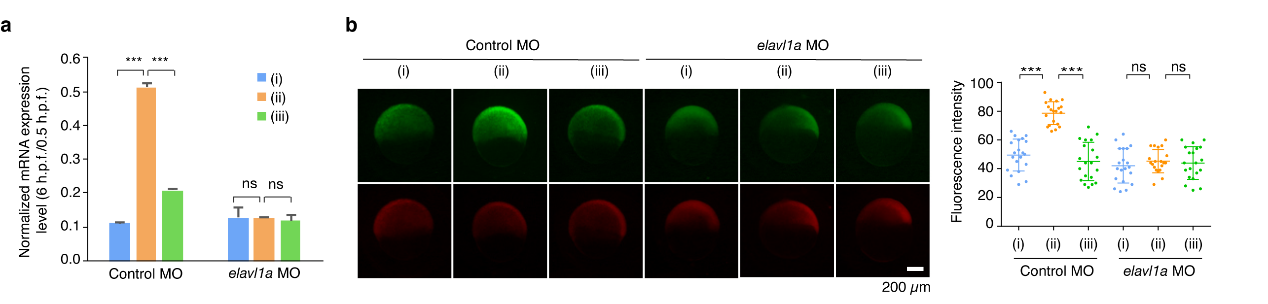
**

**Fig. S6 Full-length 3’ UTR reporter gene assay shows Elavl1a regulates RNA stability in a structure-dependent fashion.** (a) The relative mRNA level (6 h.p.f. versus 0.5 h.p.f.) of reporter genes with different structural contexts of Elavl1a binding motif in control and *elavl1a* morphants, n = 3 for each group, Error bars, mean ± s.d, *P* values were determined by two-sided Student’s *t*-test. (b) The protein level of reporter gene with different structural contexts of Elavl1a binding motif at 6 h.p.f. in control and *elavl1a* morphants determined by GFP fluorescence signal observation. Some pictures of representative embryos were shown. Quantitative fluorescence intensity was shown on the right, n = 20 for each group. Error bars, mean ± s.d. *P* values were determined by two-sided Student’s *t*-test.

**
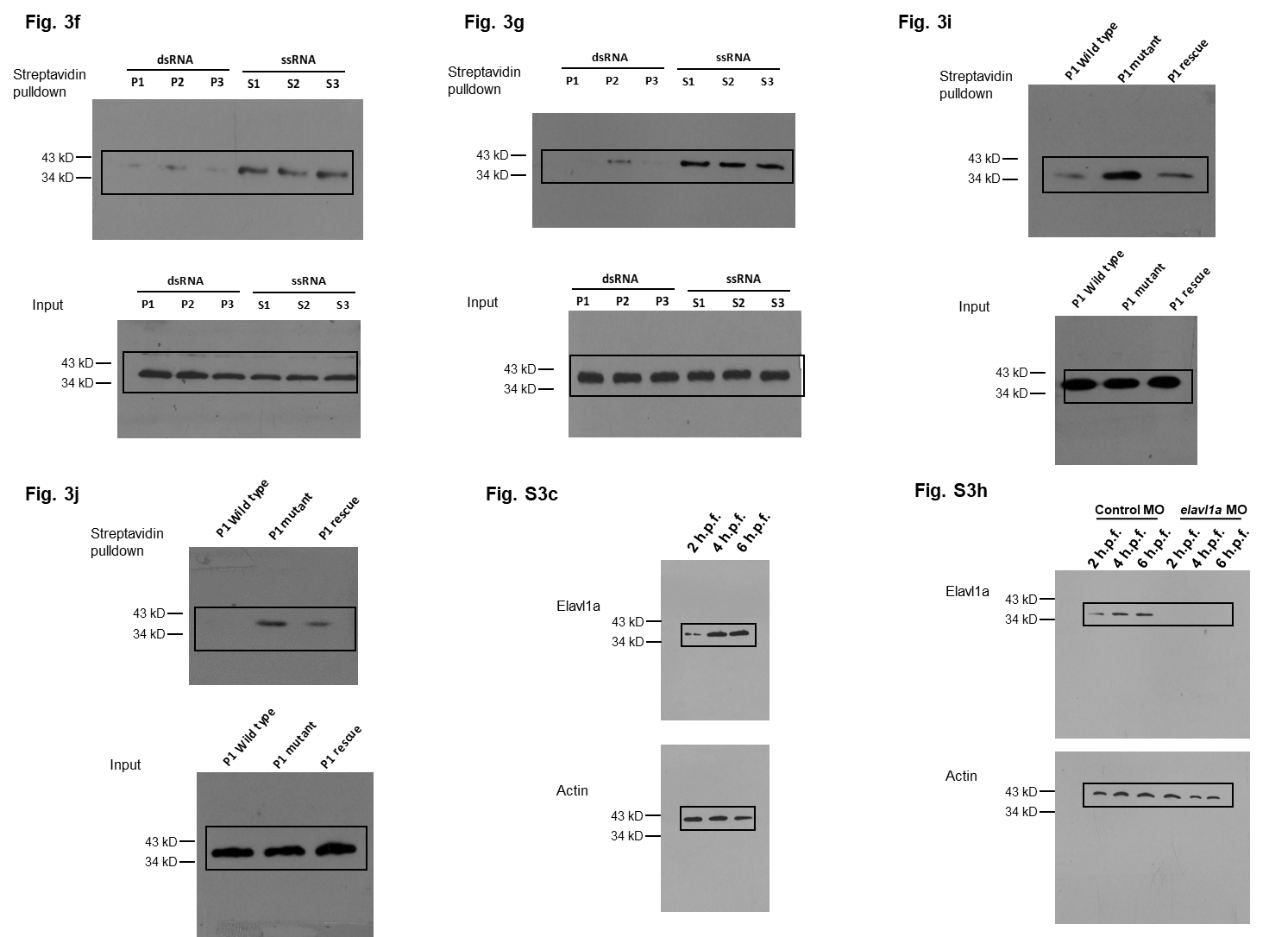
**

**Fig. S7 Full blots of figures**
