# Supplementary material for: RNA structural dynamics regulate early embryogenesis through controlling transcriptome fate and function
Source: Genome Biol. 2020 May 18;21:120. doi: 10.1186/s13059-020-02022-2 (PMC7236375; doi:10.1186/s13059-020-02022-2)
Supplement: Supplementary file 11 — Review history. [file 13059_2020_2022_MOESM11_ESM.docx]

Review History

**First round of review**

**Reviewer 1**

**Are you able to assess all statistics in the manuscript, including the appropriateness of statistical tests used?**

No, I do not feel adequately qualified to assess the statistics.

**Comments to author:**

The study by Shi and colleagues describes the genome-wide RNA four nucleotide based structural landscape in early zebrafish embryogenesis. Applying the method they developed, icSHAPE, they discovered that a majority of maternal mRNAs displayed structurally variable regions mainly in their 3'UTR region. Furthermore, a substantial proportion of these structurally variable regions contained binding sites of proteins regulating mRNA stability, one of the most predominant being Elavl1a. Their analyses on Elavl1a revealed its requirement in early embryogenesis through stabilizing maternal mRNAs by binding to its cis element in a single-stranded form. They also showed that this stabilizing role is regulated by structural changes during maternal to zygotic transition leading to degradation of the maternal mRNA.

Overall, this is a well-conducted study employing rigorous methods to analyze global RNA structure and the role of Elavl1a in structure-dependent regulation of RNA stability in vivo. RNA structure has been largely overlooked within the context of early embryogenesis and therefore the information gained from this study opens new avenues of investigations into the function and mechanism of RNA structure in further refining developmental processes. The case of Elavl1a is a good example of such mechanism which the authors have nicely shown through their functional analyses. Following the history of revisions in Molecular Cell, the manuscript has undergone much improvement and, despite the removal of the Ddx3 part, is sufficiently novel and contributes significant advance in the field of RNA structure which would be of outstanding interest to the readership of Genome Biology.

I have several minor suggestions for improvement:

1. Figure 4A: what does the x axis represent?

2. In the introduction, the authors referred to the observation that "maternal transcripts undergo posttranscriptional and translational regulation including selective elongation of the poly(A) tail" and "The poly(A) tails of maternal RNAs remain short immediately after fertilization and then gradually elongate during the cleavage stage, which might coincide with translation". However the reference they cited doesn't seem to be correct. Perhaps they meant to cite Winata et al., Development 2018?

3. The authors showed that structurally variable regions in the 3'UTR contain known binding sites of RBPs and cited examples of CPEBs, Pumilio, and Elavl1. It would be interesting to check whether these structurally variable regions also include that of the dorsal localization elements found in Nodal transcripts which is recognized by Ybx1 translational repressor (Zaucker et al. NAR 2018) which is one of the first examples of structure-based cis-regulation of RNA translation demonstrated in the zebrafish.

4. The definition of maternal stable and maternal decay mRNAs was based on the expression increase or decrease of expression levels between 2 hpf and 6 hpf. Although this would be accurate for a majority of the maternal transcripts, there are certain exceptions which would include those genes whose zygotic transcript is expressed starting from ZGA. Perhaps the authors could further refine their gene list by comparing it with Harvey et al. Development 2013 which uses maternal vs. paternal SNPs to accurately distinguish maternal from zygotic transcripts.

**Reviewer 2**

**Are you able to assess all statistics in the manuscript, including the appropriateness of statistical tests used?**

Yes, and I have assessed the statistics in my report.

**Comments to author:**

In this manuscript, the authors used icSHAPE to probe RNA accessibility in zebrafish embryos at various developmental stages. Potential differences were found that are enriched at the 3'UTR of mRNA. An interesting finding is that the protein Elavl1, appears to bind mRNA in a secondary structure dependent manner to regulate maternal RNA stability in various developmental stages. The manuscript is suitable for publication after the authors perform some important controls needed to support their main claims.

Major:

1. The authors interpret an increase or decrease in icSHAPE reactivity as "a structure opening" or "closing". First, it is not clear that the observed global changes in SHAPE reactivity in Figure 1D are statistically. Second, changes in reactivity could reflect differences in the binding of protein to the same RNA structure at different stages (Spitale et al., 2013). RNA binding proteins could decrease reactivity by directly protecting the same unchanging RNA structure - and reactivity could increase owing to weaker protein binding. The authors need to consider and ideally rule out this possibility and adjust their claims accordingly.

2. In general, the relationship among protein binding, RNA structure change and SHAPE reactivity change is not clear. The authors prefer the model in which the SHAPE reactivity change originates from an "RNA structure-switch", which modulates protein binding. But differences in protein binding to the same RNA structure due to for example different protein expression levels may also cause changes in SHAPE reactivity either due to protection of the RNA or because protein binding induces a structure with altered reactivity. These factors appear to be important when considering the interesting Elavl1 protein. Although the authors clearly showed that Elavl1 binds to ssRNA, it is important to establish that changes in SHAPE reactivity are primarily driven by inherent changes in RNA structure unrelated to protein binding.

3. The authors seem to imply that Elavl1a reversibly binds to RNA depending on whether the RNA is more or less structured (Figure 6E). For example, in Figure 4D, mylipa is less structured and can be bound by Elavl1 in 4.h.p.f, while mylipa is more structured in 6.h.p.f, thus repelling Elavl1 and resulting in degradation. The changes in RNA structure and protein behavior appears dramatic and need explanation. How does the free energy of protein binding compare to RNA structural switch? What are the other factors that may promote the RNA structural switch?

Minor:

1. Line 114 a "cutoff of Δreactivity score> 0.2" , and on line 123, "the average Δreactivity score> 0.05" are used to assess the significant structural change for a single nucleotide and a 10 nt sliding window. How these cutoffs were chosen needs to be stated in the methods section.

2. Line 93-94, "evaluate the concordance of icSHAPE reactivity between the conserved secondary structures of dgcr8" should be "evaluate the concordance between icSHAPE reactivities and the conserved secondary structure of dgcr8".

**Authors Response**

**Point-by-point responses to the reviewers’ comments:**

Point-by-point responses to reviewers’ comments

Reviewer #1: The study by Shi and colleagues describes the genome-wide RNA four nucleotide based structural landscape in early zebrafish embryogenesis. Applying the method they developed, icSHAPE, they discovered that a majority of maternal mRNAs displayed structurally variable regions mainly in their 3'UTR region. Furthermore, a substantial proportion of these structurally variable regions contained binding sites of proteins regulating mRNA stability, one of the most predominant being Elavl1a. Their analyses on Elavl1a revealed its requirement in early embryogenesis through stabilizing maternal mRNAs by binding to its cis element in a single-stranded form. They also showed that this stabilizing role is regulated by structural changes during maternal to zygotic transition leading to degradation of the maternal mRNA.

Overall, this is a well-conducted study employing rigorous methods to analyze global RNA structure and the role of Elavl1a in structure-dependent regulation of RNA stability in vivo. RNA structure has been largely overlooked within the context of early embryogenesis and therefore the information gained from this study opens new avenues of investigations into the function and mechanism of RNA structure in further refining developmental processes. The case of Elavl1a is a good example of such mechanism which the authors have nicely shown through their functional analyses. Following the history of revisions in Molecular Cell, the manuscript has undergone much improvement and, despite the removal of the Ddx3 part, is sufficiently novel and contributes significant advance in the field of RNA structure which would be of outstanding interest to the readership of Genome Biology.

*Response: We thank the reviewer for the thoughtful advice. We have followed the advice and improved the manuscripts with additional analysis.*

I have several minor suggestions for improvement:

1. Figure 4A: what does the x axis represent ?

*Response: We apologize for missing this label. The x-axis of Fig 4A represents the nucleotide positions, centered at the Elavl1a binding site (the following Revision Figure 1, also included in the revision as the new Figure 4A).*

*Revision Figure 1. Average icSHAPE reactivity change (4 h.p.f. and 6 h.p.f.) at the Elavl1a binding site of the two groups of transcripts.*

2. In the introduction, the authors referred to the observation that "maternal transcripts undergo posttranscriptional and translational regulation including selective elongation of the poly(A) tail" and "The poly(A) tails of maternal RNAs remain short immediately after fertilization and then gradually elongate during the cleavage stage, which might coincide with translation". However the reference they cited doesn't seem to be correct. Perhaps they meant to cite Winata et al., Development 2018 ?

*Response: We agree and have cited the paper (Winata et al., Development 2018. line 56).*

3. The authors showed that structurally variable regions in the 3'UTR contain known binding sites of RBPs and cited examples of CPEBs, Pumilio, and Elavl1. It would be interesting to check whether these structurally variable regions also include that of the dorsal localization elements found in Nodal transcripts which is recognized by Ybx1 translational repressor (Zaucker et al. NAR 2018) which is one of the first examples of structure-based cis-regulation of RNA translation demonstrated in the zebrafish.

*Response: Thanks for this thoughtful suggestion. We agree that the dorsal localization element (DLE) is a good example of structure-based regulation.*

*We searched for DLE in the zebrafish structurome and found that many transcripts contain the motif. However, it is not enriched in the structurally variable regions. And unfortunately, the RNA structure coverage for the four Nodal transcripts (ndr1, ndr2, lft1or lft2) at the DLE motif positions is poor, probably due to the relatively low expression level in zebrafish early embryos.*

*We analyzed all the transcripts with structurally variable regions in the 3'UTRs that contain the DLE structure motif (a consensus “AGCAC” sequence in a hairpin/stem-loop in 3’UTR, (Gilligan et al., 2011; Zaucker et al., 2018)). We found that the genes are enriched with the functions of “protein localization”, “protein transport”, etc. (see the following Revision Figure 2A-B). Some examples are shown below (Revision Fig 2C). This finding is consistent with a previous study (Gilligan et al., 2011), furthermore, our findings could help to find the potential protein regulators and understand how RNA structural changes participate in the regulation.*

*We have included these results as the new Figure S2H and TableS4 in the revised manuscript.*

*Revision Figure 2. The dorsal localization element (DLE) in RNA structurally variable regions.*

*(A) The number of DLE occurrences in RNA structurally variable regions and the number of host transcripts harboring the element in their 3'UTRs.*

*(B) Gene set enrichment analysis of the transcripts that contain a DLE motif in structurally variable regions.*

*(C) RNA structural models of the DLE motifs (with flanking sequences) in the structurally variable regions of the 3'UTRs of ctbp2a and emc2 mRNA.*

4. The definition of maternal stable and maternal decay mRNAs was based on the expression increase or decrease of expression levels between 2 hpf and 6 hpf. Although this would be accurate for a majority of the maternal transcripts, there are certain exceptions which would include those genes whose zygotic transcript is expressed starting from ZGA. Perhaps the authors could further refine their gene list by comparing it with ， which uses maternal vs. paternal SNPs to accurately distinguish maternal from zygotic transcripts.

*Response: We agree and thank the reviewer for the suggestion.*

*We compared our maternal gene set with Harvey et al. Development 2013 (Harvey et al., 2013), and found that 3182 genes of our gene set could distinguish maternal or zygotic expression by using maternal vs. paternal SNPs. Among these, 859 genes were only maternally expressed, 143 genes were only zygotically expressed, and 2180 genes had both maternal and zygotic expression.*

*94.3% (810/859) of the maternal-only genes decayed between 2 h.p.f. and 6 h.p.f., and the other 4.9% (42/859) genes were stable (Revision Figure 3A). Interestingly, 65.3% (1424/2180) genes with maternal and zygotic expression decayed between 2 h.p.f. and 6 h.p.f., and the other 32.6% (710/2180) were stable (Revision Fig 3B). In another word, even with zygotic contributions, the expression levels of most of these genes still decreased during zebrafish embryogenesis, i.e., maternal decay genes.*

*After narrowing down the definition of maternal gene set by maternal vs. paternal SNPs, 274 out of 817 group I genes were categorized into the “more structural” group and 69% (187/274) showed maternal decay, with 24 and 163 in maternal-only and maternal-and-zygotic group, respectively (Revision Figure 3C, left). However, in the “less structural” genes, 62% (38/61) were maternally stable with 1 and 37 in maternal-only and maternal-and-zygotic group, respectively (Revision Figure 3C, right). These results are very similar to that we obtained from the much bigger maternal gene set defined by gene expression change.*

*We have included these results as the new Figure S4C-E and TableS8 in the revised manuscript.*

*Revision Figure 3. Maternal decay genes are enriched in the “more structural” group and maternal stable genes are enriched in the “less structural” group.*

*(A-B) Overlap of maternal decay and stable genes defined by only the change of RNA abundance and maternal-only (A) and maternal-and-zygotic (B) genes by using maternal vs. paternal SNPs.*

*(C) Pie chart depicting the proportion of maternal decay genes (including both the maternal-only and the maternal-and-zygotic groups) in transcripts with more structural Elavl1a binding sites, and the proportion of maternal stable genes (both maternal-only and maternal-and-zygotic) in transcripts with less structural Elavl1a binding sites during the period of 4 h.p.f. to 6 h.p.f..*

Reviewer #2: In this manuscript, the authors used icSHAPE to probe RNA accessibility in zebrafish embryos at various developmental stages. Potential differences were found that are enriched at the 3'UTR of mRNA. An interesting finding is that the protein Elavl1, appears to bind mRNA in a secondary structure dependent manner to regulate maternal RNA stability in various developmental stages. The manuscript is suitable for publication after the authors perform some important controls needed to support their main claims.

*Response: We highly appreciate the reviewer for the enthusiastic assessment and also the thoughtful comments. We have followed the comments and improved the manuscripts with additional experiments and analysis. In the following, we address all the questions in a point-by-point way.*

Major:

1. The authors interpret an increase or decrease in icSHAPE reactivity as "a structure opening" or "closing". First, it is not clear that the observed global changes in SHAPE reactivity in Figure 1D are statistically. Second, changes in reactivity could reflect differences in the binding of protein to the same RNA structure at different stages (Spitale et al., 2013). RNA binding proteins could decrease reactivity by directly protecting the same unchanging RNA structure - and reactivity could increase owing to weaker protein binding. The authors need to consider and ideally rule out this possibility and adjust their claims accordingly .

*Response: We thank the reviewer for the thoughtful advice. First of all, we performed the two-sided paired t-test and found that the global changes in SHAPE reactivity are significant except the period of 4 h.p.f. to 6 h.p.f.. We have labeled the significance values in the Revised Figure 1D.*

*We agree it is a critical issue whether the observed structure change is true or not. As the reviewer pointed out, it also might be due to the changes of protein bindings that affect the effectiveness of structure probing. However, we have three lines of evidence that it is not likely the case:*

*1), We have performed in vivo mRBPs (mRNA binding proteins) pulldown by using 0/0.4/4 h.p.f. embryos, and observed no global mRBPs binding changes among these stages (see the following Revision Figure 4, also included as the Revised Figure S1I).*

*Revision Figure 4. Cumulative distribution of the log2 fold of change of LFQ intensity of mRBPs between UV+ and UV- samples at 0, 0.4, 4 h.p.f. Statistical significances were calculated by the two-sided Kolmogorov-Smirnov test. The exact p-value is 0.21 for 0 h.p.f. vs 0.4 h.p.f., 0.17 for 0.4 h.p.f. vs 4 h.p.f., and 0.71 for 0 h.p.f. vs 4 h.p.f..*

*2). We systematically analyzed the icSHAPE reactivity score distribution for a very large dataset of RBP binding sites from 23 iCLIP studies in zebrafish (Vejnar et al., 2019), and compared it with that of the random nucleotides on the whole transcripts. If RBP binding generally protects RNA from the probing reaction of NAI-N3, the icSHAPE reagent, we would hope to see that the icSHAPE reactivity scores are lower at these RBP binding sites compared to random positions. However, we see very small reactivity score differences between the two datasets, suggesting that RBP binding does not necessarily block NAI-N3 modification (see the following Revision Figure 5A, also included as the Revised Figure S1J). We even repeat the analysis with human structurome data and much more CLIP-seq data (65 studies in total) and confirmed the observation (Revision Figure 5B).*

*Revision Figure 5. Box plot shows very small differences between the icSHAPE reactivity scores at RBP binding sites and random positions on the whole transcripts in zebrafish (A) and human (B).*

*3), the above observation is likely due to the small size of NAI-N3, which has minimized spatial hindrance by RBPs and/or other molecules that interact with RNA. This is demonstrated by the structure probing experiments of the ribosomal RNAs in the ribosome complex as in the 2013 and 2015 research articles by Spitale et al. (Spitale et al., 2013; Spitale et al., 2015). As can be seen in the following Revision Figure 6 adapted from Spitale et. al 2013, the gel bands (left) shows that most reactive bases (right) can be well probed. In other words, can be accessed by the chemical, even though the ribosome structure is very compact and many nucleotides are buried inside the structure.*

*Revision Figure 6. Gel shifts of NAI-N3 probing and three-dimensional model with NAI-N3 modifications for the S. cerevisiae 5S rRNA (Adapted from Spitale et. al 2013)*

*Another example of RNA-protein complex is from the Rbfox2 protein-RNA interaction NMR structure in the same paper (see the following Revision Figure 7 adapted from Spitale et. al 2015). Panel a shows the structural model of the complex and b shows the in vivo and in vitro structure differences. Note that the in vitro structures are structures without protein binding, while the in vivo structures are those with protein binding in cells. Here one can see that Rbfox binding actually “flips out” the 2'-hydroxyl groups of the cognate RNA motif to the solvent exposed surface of the complex (with the exception of C3 which is now buried), and the icSHAPE signal for these residues in vivo are larger than that in vitro, suggesting that protein binding does not block the 2’-hydroxyl groups from NAI-N3 access.*

*Revision Figure 7. Three-dimensional model of Rbfox binding to RNA and in vivo and in vitro icSHAPE reactivity score differences at Rbfox binding sites.*

2. In general, the relationship among protein binding, RNA structure change and SHAPE reactivity change is not clear. The authors prefer the model in which the SHAPE reactivity change originates from an "RNA structure-switch", which modulates protein binding. But differences in protein binding to the same RNA structure due to for example different protein expression levels may also cause changes in SHAPE reactivity either due to protection of the RNA or because protein binding induces a structure with altered reactivity. These factors appear to be important when considering the interesting Elavl1 protein. Although the authors clearly showed that Elavl1 binds to ssRNA, it is important to establish that changes in SHAPE reactivity are primarily driven by inherent changes in RNA structure unrelated to protein binding .

*Response: Thanks for this insightful comment. We agree that overall it is difficult to distinguish the driving force between structural changes and RBP binding changes. For the case of Elavl1 binding, we did the following additional experiments to show that it is more likely the structural changes lead to Elavl1 binding changes.*

*First, to explore whether or not in vivo Elavl1a expression influences the RNA structure at Elavl1a binding sites, we performed icSHAPE assays by using wild-type (WT) and Elavl1a knockdown (by elavl1a MO injection) samples at stage of 4 h.p.f. and 6 h.p.f.. The meta-gene analysis of the structural profile at Elavl1a binding sites showed that these binding sites are more accessible than the nonbinding sites (with the same sequence motif), even in the condition of Elavl1a deficiency (Revision Figure 8, also Revised Figure S3I and S3J).*

*Revision Figure 8. Averaged icSHAPE reactivity profiles in 4 h.p.f. (A) and 6 h.p.f. (B) between Elavl1a binding sites identified by iCLIP and non-binding sites with the same sequence motif, in the wild-type (WT) and Elavl1a deficiency (MO) embryos.*

*Moreover, we analyzed the structure change at the Elavl1a binding sites that form more structures between 4 h.p.f. and 6 h.p.f.. We found that the structure changes are similar between the wild-type (WT) and Elavl1a deficiency (MO) embryos, suggesting that these changes is independent of the Elavl1a binding. (Revision Figure 9, also Revised Figure S4F).*

*Taken together, these results suggest that it is more likely that the RNA structural changes are not due to Elavl1a binding, but originate from an "RNA structure-switch" and modulate the protein binding.*

*Revision Figure 9. Average icSHAPE reactivity scores around the Elavl1a binding sites that form more structure between 4 h.p.f. and 6 h.p.f., in WT and elavl1a MO samples. Statistical significances were calculated by the two-sided unpaired Student’s t test.*

3. The authors seem to imply that Elavl1a reversibly binds to RNA depending on whether the RNA is more or less structured (Figure 6E). For example, in Figure 4D, mylipa is less structured and can be bound by Elavl1 in 4.h.p.f, while mylipa is more structured in 6.h.p.f, thus repelling Elavl1 and resulting in degradation. The changes in RNA structure and protein behavior appears dramatic and need explanation. How does the free energy of protein binding compare to RNA structural switch? What are the other factors that may promote the RNA structural switch ?

*Response: We appreciate this thoughtful comment and have investigated the energy basis of RNA structural switch and protein binding following the suggestion.*

*First, we estimated the free energy of protein binding and RNA structural switch. We used PredPRBA (Deng et al., 2019) to calculate free energy of Elavl1a-RNA binding, by using five crystal structures of the human Elavl1/RNA complex (Pabis et al., 2019; Ripin et al., 2019; Wang et al., 2013). The average of free energy of Elavl1a-RNA binding is about -8.66 kcal/mol (Revision figure 10A). However, the free energy of RNA structural switch between the two RNA conformation is only about -0.6 kcal/mol (-15.4 kcal/mol - -14.8 kcal/mol) (Revision figure 10B), confirming that RNA structure is highly dynamic. Thus, it is highly likely that RNA structures are perturbed by cellular factors and consequently affect Elavl1a binding.*

*Many factors inside a living cell, such as temperature, ion strength, crowding effect, cellular energy state, and the binding of RBPs including ATP-dependent RNA helicases, chaperone proteins and others, can affect and change RNA structures (Lewis et al., 2017). As Elavl1a itself does not seem affecting the RNA structure at the set of Elavl1a binding sites investigated in this study (see the above response to point 2), we think that other cellular factors may promote the proposed RNA structural switch. We have included some discussions in the revised manuscript.*

*Revision Figure 10. Free energy of protein binding and RNA structural switch*

*(A) Free energy of Elavl1a binding in five crystal structures calculated by PredPRBA.*

*(B) The RNA structure model and the free energy of Elavl1a binding site in the 3’UTR of mylipa at 4 and 6 h.p.f.. Models were constructed by using RNAstructure with icSHAPE reactivity scores as constraint.*

Minor:

1. Line 114 a " cutoff of Δreactivity score> 0.2" , and on line 123, "the average Δreactivity score> 0.05" are used to assess the significant structural change for a single nucleotide and a 10 nt sliding window. How these cutoffs were chosen needs to be stated in the methods section .

*Response: We apologize for missing the details and have updated the Methods.*

*For individual nucleotides, we chose the same cutoff 0.2 used in our previous study (Sun et al., 2019), where the cutoff was estimated to roughly represent the 5% most significant structural changes. Additionally, here we performed icSHAPE on folded spike-in RNAs, and found that more than 98.5% base with differential icSHAPE reactivity score lower than 0.2 between replicates (Revision Fig 11A). So, we think the cutoff of Δreactivity score> 0.2 is enough to filter out most technical noise yet to keep true structural change for a single nucleotide.*

*For 10-nt sliding windows, we used the similar strategy to choose the cutoff, by ranking all windows according to the average Δreactivity score. About 95% windows have the average Δreactivity score lower than 0.05. For every window, we also calculated the significance of the difference by using the two-sided paired Student’s t-test. To be regarded as a significant structural change, we require the P value <0.05. In sum, the average Δreactivity score> 0.05 and and P value <0.05 (two-sided paired Student’s t test) were used to assess the significant structural change for a 10 nt sliding window. The whole process is very much like the differential gene expression analysis (Revision Fig 11B, also Revised Figure S2B and S2C).*

*Revision Figure 11. Selection of cutoffs of Δreactivity score for individual nucleotides and 10-nt sliding windows.*

*(A) Cumulative distribution of differential icSHAPE reactivity scores of the spike-in RNAs between replicates at single nucleotide resolution. Inset is the scatter plot showing icSHAPE reactivity scores between replicates.*

*(B) Volcano plot shows the distribution of averaged differential icSHAPE reactivity scores and log10 P values for pairwise 10-nt sliding windows of the spike-in RNAs between replicates. Statistical significances were calculated using the two-sided paired Student’s t-test.*

2. Line 93-94, "evaluate the concordance of icSHAPE reactivity between the conserved secondary structures of dgcr8" should be "evaluate the concordance between icSHAPE reactivities and the conserved secondary structure of dgcr8".

*RESPONSE: We thank the reviewer for noting this and have updated the text (line 94-95).*

*References*

*Deng, L., Yang, W., and Liu, H. (2019). PredPRBA: Prediction of Protein-RNA Binding Affinity Using Gradient Boosted Regression Trees. Frontiers in genetics 10, 637.*

*Gilligan, P.C., Kumari, P., Lim, S., Cheong, A., Chang, A., and Sampath, K. (2011). Conservation defines functional motifs in the squint/nodal-related 1 RNA dorsal localization element. Nucleic Acids Res 39, 3340-3349.*

*Harvey, S.A., Sealy, I., Kettleborough, R., Fenyes, F., White, R., Stemple, D., and Smith, J.C. (2013). Identification of the zebrafish maternal and paternal transcriptomes. Development (Cambridge, England) 140, 2703-2710.*

*Lewis, C.J., Pan, T., and Kalsotra, A. (2017). RNA modifications and structures cooperate to guide RNA-protein interactions. Nat Rev Mol Cell Biol 18, 202-210.*

*Pabis, M., Popowicz, G.M., Stehle, R., Fernandez-Ramos, D., Asami, S., Warner, L., Garcia-Maurino, S.M., Schlundt, A., Martinez-Chantar, M.L., Diaz-Moreno, I., et al. (2019). HuR biological function involves RRM3-mediated dimerization and RNA binding by all three RRMs. Nucleic Acids Res 47, 1011-1029.*

*Ripin, N., Boudet, J., Duszczyk, M.M., Hinniger, A., Faller, M., Krepl, M., Gadi, A., Schneider, R.J., Sponer, J., Meisner-Kober, N.C., et al. (2019). Molecular basis for AU-rich element recognition and dimerization by the HuR C-terminal RRM. Proceedings of the National Academy of Sciences of the United States of America 116, 2935-2944.*

*Spitale, R.C., Crisalli, P., Flynn, R.A., Torre, E.A., Kool, E.T., and Chang, H.Y. (2013). RNA SHAPE analysis in living cells. Nature chemical biology 9, 18-20.*

*Spitale, R.C., Flynn, R.A., Zhang, Q.C., Crisalli, P., Lee, B., Jung, J.W., Kuchelmeister, H.Y., Batista, P.J., Torre, E.A., Kool, E.T., et al. (2015). Structural imprints in vivo decode RNA regulatory mechanisms. Nature 519, 486-490.*

*Sun, L., Fazal, F.M., Li, P., Broughton, J.P., Lee, B., Tang, L., Huang, W., Kool, E.T., Chang, H.Y., and Zhang, Q.C. (2019). RNA structure maps across mammalian cellular compartments. Nature structural & molecular biology 26, 322-330.*

*Vejnar, C.E., Abdel Messih, M., Takacs, C.M., Yartseva, V., Oikonomou, P., Christiano, R., Stoeckius, M., Lau, S., Lee, M.T., Beaudoin, J.D., et al. (2019). Genome wide analysis of 3' UTR sequence elements and proteins regulating mRNA stability during maternal-to-zygotic transition in zebrafish. Genome research 29, 1100-1114.*

*Wang, H., Zeng, F., Liu, Q., Liu, H., Liu, Z., Niu, L., Teng, M., and Li, X. (2013). The structure of the ARE-binding domains of Hu antigen R (HuR) undergoes conformational changes during RNA binding. Acta crystallographica Section D, Biological crystallography 69, 373-380.*

*Zaucker, A., Nagorska, A., Kumari, P., Hecker, N., Wang, Y., Huang, S., Cooper, L., Sivashanmugam, L., VijayKumar, S., Brosens, J., et al. (2018). Translational co-regulation of a ligand and inhibitor by a conserved RNA element. Nucleic Acids Res 46, 104-119.*

**Second round of review**

**Reviewer 2**

Accept
